# LDLR dysfunction induces LDL accumulation and promotes pulmonary fibrosis

**DOI:** 10.1002/ctm2.711

**Published:** 2022-01-26

**Authors:** Xiangguang Shi, Yahui Chen, Qingmei Liu, Xueqian Mei, Jing Liu, Yulong Tang, Ruoyu Luo, Dayan Sun, Yanyun Ma, Wenyu Wu, Wenzhen Tu, Yinhuan Zhao, Weihong Xu, Yuehai Ke, Shuai Jiang, Yan Huang, Rui Zhang, Lei Wang, Yuanyuan Chen, Jingjing Xia, Weilin Pu, Honglin Zhu, Xiaoxia Zuo, Yisha Li, Jinhua Xu, Fei Gao, Dong Wei, Jingyu Chen, Wenguang Yin, Qingwen Wang, Huaping Dai, Libing Yang, Gang Guo, Jimin Cui, Nana Song, Hejian Zou, Shimin Zhao, Jörg H.W. Distler, Li Jin, Jiucun Wang

**Affiliations:** ^1^ Department of Dermatology, Huashan Hospital and State Key Laboratory of Genetic Engineering, School of Life Sciences Fudan University Shanghai P. R. China; ^2^ Human Phenome Institute and Collaborative Innovation Center for Genetics and Development Fudan University Shanghai P. R. China; ^3^ Division of Rheumatology Huashan hospital, Fudan University Shanghai P. R. China; ^4^ MOE Key Laboratory of Contemporary Anthropology, Department of Anthropology and Human Genetics, School of Life Sciences Fudan University Shanghai P. R. China; ^5^ Institute for Six‐sector Economy Fudan University Shanghai P. R. China; ^6^ Division of Rheumatology Shanghai TCM‐Integrated Hospital Shanghai P. R. China; ^7^ The Clinical Laboratory of Tongren Hosipital Shanghai Jiaotong University Shanghai P. R. China; ^8^ Department of Pathology and Pathophysiology Zhejiang University School of Medicine Hangzhou Zhejiang Province P. R. China; ^9^ Department of Internal Medicine 3 and Institute for Clinical Immunology University of Erlangen Nuremberg Germany; ^10^ Department of Rheumatology, Xiangya Hospital Central South University Changsha Hunan Province P. R. China; ^11^ Wuxi Lung Transplant Center Wuxi People's Hospital affiliated to Nanjing Medical University Wuxi P. R. China; ^12^ State Key Laboratory of Respiratory Disease, National Clinical Research Center for Respiratory Disease, Guangzhou Institute of Respiratory Health The First Affiliated Hospital of Guangzhou Medical University Guangzhou Guangdong P. R. China; ^13^ Rheumatology and Immunology Department Peking University Shenzhen Hospital Shenzhen P. R. China; ^14^ Department of Pulmonary and Critical Care Medicine, China‐Japan Friendship Hospital; National Clinical Research Center for Respiratory Diseases, Institute of Respiratory Medicine Chinese Academy of Medical Science Beijing P. R. China; ^15^ School of Medicine Tsinghua University Beijing P. R. China; ^16^ Department of Rheumatology and Immunology Yiling Hospital Affiliated to Hebei Medical University Shijiazhuang Hebei Province P. R. China; ^17^ Department of Nephrology, Zhongshan Hospital, Fudan University Fudan Zhangjiang Institute Shanghai P. R. China; ^18^ Institute of Rheumatology, Immunology and Allergy Fudan University Shanghai P. R. China; ^19^ Institute of Metabolism and Integrative Biology Fudan University Shanghai P. R. China; ^20^ Research Unit of Dissecting the Population Genetics and Developing New Technologies for Treatment and Prevention of Skin Phenotypes and Dermatological Diseases (2019RU058) Chinese Academy of Medical Sciences Shanghai P. R. China

**Keywords:** apoptosis, combination treatment, LDL, LDLR, pulmonary fibrosis

## Abstract

Treatments for pulmonary fibrosis (PF) are ineffective because its molecular pathogenesis and therapeutic targets are unclear. Here, we show that the expression of low‐density lipoprotein receptor (LDLR) was significantly decreased in alveolar type II (ATII) and fibroblast cells, whereas it was increased in endothelial cells from systemic sclerosis‐related PF (SSc‐PF) patients and idiopathic PF (IPF) patients compared with healthy controls. However, the plasma levels of low‐density lipoprotein (LDL) increased in SSc‐PF and IPF patients. The disrupted LDL–LDLR metabolism was also observed in four mouse PF models. Upon bleomycin (BLM) treatment, *Ldlr*‐deficient (*Ldlr*−/−) mice exhibited remarkably higher LDL levels, abundant apoptosis, increased fibroblast‐like endothelial and ATII cells and significantly earlier and more severe fibrotic response compared to wild‐type mice. In vitro experiments revealed that apoptosis and TGF‐β1 production were induced by LDL, while fibroblast‐like cell accumulation and ET‐1 expression were induced by *LDLR* knockdown. Treatment of fibroblasts with LDL or culture medium derived from LDL‐pretreated endothelial or epithelial cells led to obvious fibrotic responses in vitro. Similar results were observed after LDLR knockdown operation. These results suggest that disturbed LDL–LDLR metabolism contributes in various ways to the malfunction of endothelial and epithelial cells, and fibroblasts during pulmonary fibrogenesis. In addition, pharmacological restoration of LDLR levels by using a combination of atorvastatin and alirocumab inhibited BLM‐induced LDL elevation, apoptosis, fibroblast‐like cell accumulation and mitigated PF in mice. Therefore, LDL–LDLR may serve as an important mediator in PF, and LDLR enhancing strategies may have beneficial effects on PF.

## Graphical abstract

Pulmonary fibrosis (PF) is a devastating lung disorder of unknown etiology. We show that disrupted LDL‐LDLR metabolism in human and mouse PF, and then investigated their contributions and underlying mechanisms for PF progression from endothelial/epithelial injury to eventual fibrosis. Pharmacological restoration of LDLR expression can abolish LDL redundancy and prevent PF features.

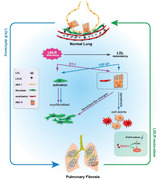

## 1 INTRODUCTION

Pulmonary fibrosis (PF) is a devastating lung disorder of unknown aetiology. Idiopathic PF (IPF) and systemic sclerosis‐related PF (SSc‐PF) are regarded as two representative lung fibrotic disorders.^1^, ^2^ IPF affects approximately 3 million people worldwide,^3^ and SSc‐PF is the main cause of death in SSc.^4^ Although the pathogeneses of different kinds of PF are not the same, including histological subtypes, disease course and survival rate, they share many common phenotypes and pathways.^5^ Excessive apoptosis of endothelial and alveolar type II (ATII) cells, increased pro‐fibrotic endothelial and ATII cells, persistent inflammation and fibroblast differentiation into myofibroblast are indispensable for the development of PF.^6^, ^7^, ^8^ Various pathways are involved in these abnormalities, especially transforming growth factor‐β (TGF‐β) and endothelin‐1 (ET‐1) pathways, which eventually lead to irreversible lung destruction.^9^, ^10^

Low‐density lipoprotein receptor (LDLR) located on type II alveolar epithelial cells (ATII) facilitate low‐density lipoprotein (LDL) particle internalization, with resultant use of endocytosed lipids and cholesterol for surfactant synthesis and secretion, which is impaired during the pathogenesis of acute lung injury.^11^, ^12^, ^13^ ATII cell injury is the initial event in the pathogenesis of PF.^14^ Massaro et al. reported that *Ldlr−*/− mice showed impaired developmental alveologenesis compared to WT mice including decreased number of alveoli, lower alveolar surface area, lower lung volume and a lower ratio of gas‐exchange surface area to gas‐exchange tissue volume.^15^ These results suggested a protective role of LDLR in lung injury and alveolar haemostasis. In addition, LDLR plays a pivotal role in clearing circulating LDL, and patients with LDLR dysfunction often have high LDL levels.^16^ A previous study has shown that LDL was increased in IPF.^17^ These results indicate that a disrupted LDL–LDLR axis may synergistically contribute to PF.

Statins are commonly prescribed medications for hypercholesterolemia and act by enhancing LDLR expression and lowering cholesterol levels, particularly LDL.^18^, ^19^ Alirocumab (pro‐protein convertase subtilisin/kexin type 9, PCSK9 antibody) prevents PCSK9‐dependent LDLR degradation and is approved for clinical use in cardiovascular diseases.^20^ The combination of atorvastatin and alirocumab is an FDA‐approved strategy to enhance the inhibitory effect on LDL production by promoting LDLR expression.^20^ In addition to reducing LDL levels, statins and anti‐PCSK9 reagents may have immunomodulatory and anti‐inflammatory properties that could be beneficial in PF.^21^, ^22^ By searching the literature investigating the association of statins with PF, we found that almost all studies showed statins are protective, but remain controversial and the mechanisms remain unclear.^23^, ^24^ However, none of these studies has focused on exploring the LDL–LDLR mechanism in statin‐taking patients or mice. Therefore, it is essential to assess the role of LDL–LDLR metabolism in PF and develop LDL–LDLR targeting therapeutic strategies.

In the present study, we show aberrant LDLR levels and increased LDL levels in PF patients. We further clarify the functional roles of LDLR and LDL in a bleomycin (BLM)‐induced PF mouse model. Combined treatment with anti‐PCSK9 and statin significantly increased LDLR expression and decreased LDL levels in mice, to a higher extent than treatment with either of the two alone. More importantly, the severity of PF was effectively reduced, while cellular apoptosis and profibrotic ATII and endothelial cells were inhibited.

## 2 RESULTS

### 2.1 Disrupted LDL–LDLR metabolism in PF patients

**FIGURE 1:**
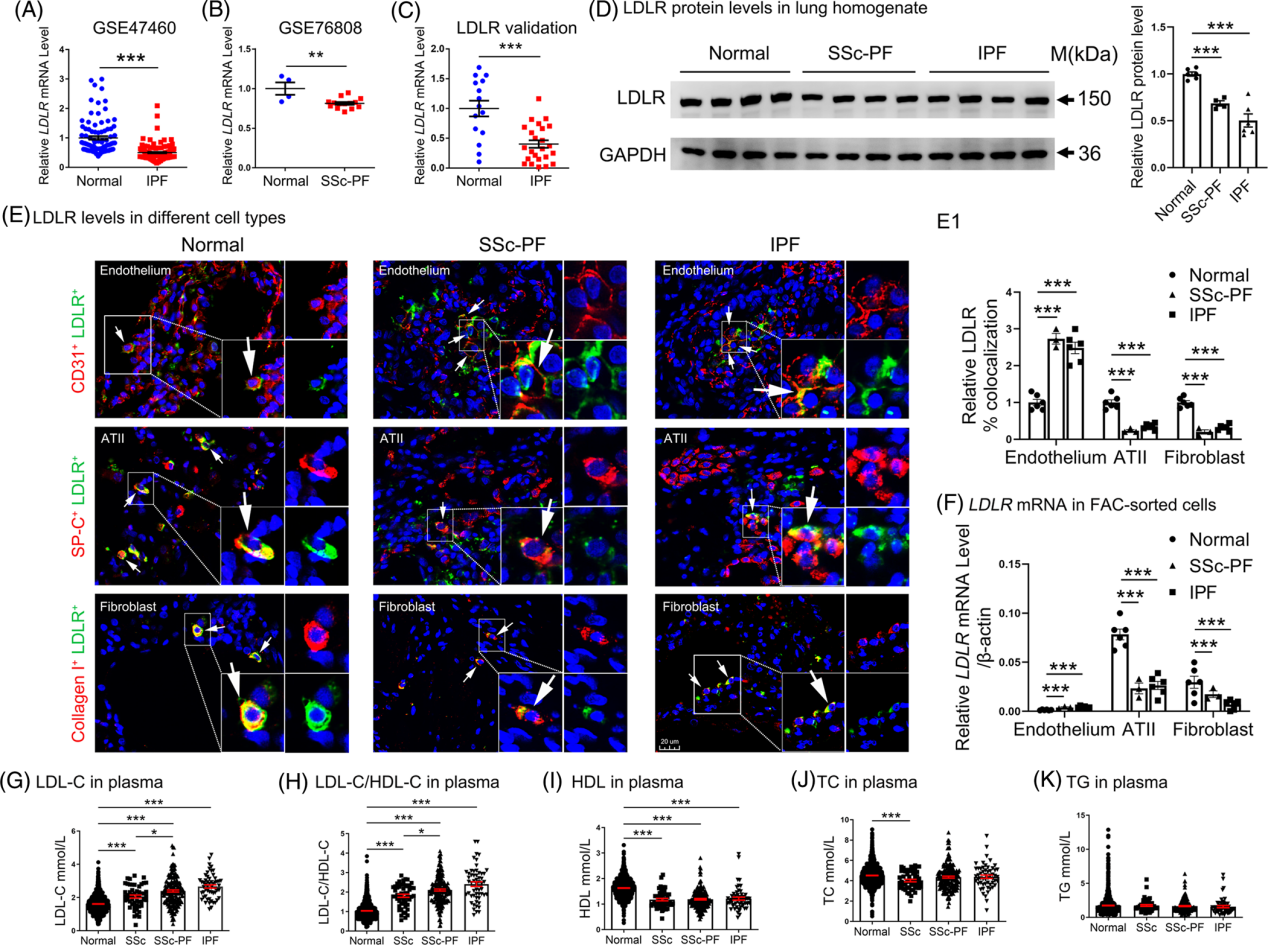
Dysregulated LDLR and increased LDL levels in PF patients. (A) *LDLR* mRNA levels in lung tissue from IPF patients (*n* = 122) and controls (*n* = 92). (B) *LDLR* mRNA levels in lung tissue from SSc‐PF patients (*n* = 14) and controls (*n* = 4). (C) *LDLR* mRNA levels in lungs from IPF patients (*n* = 24) and controls (*n* = 15). (D) Western blot analysis of LDLR protein levels in lungs of IPF patients (*n* = 6) and controls (*n* = 6). (E) Immunofluorescence staining of LDLR and CD31/SP‐C/COL1A1 in lung tissue sections from normal, SSc‐PF and IPF lungs (*n* = 6, 3, and 6, respectively). The co‐localization of LDLR and of each cell marker is expressed as a percent double positive area/sum total area of stain for each protein (N1). Scale bars: 20 μm. (F) *LDLR* levels in FAC‐sorted endothelial, ATII and fibroblast cells from normal, SSc‐PF, and IPF lungs (*n* = 6, 3 and 6, respectively) by qPCR analysis. (G–K) Lipid levels in controls, and SSc, SSc‐PF and IPF patients (*n* = 1,642, 52, 185 and 55, respectively). *p < .05, **p < .01, ***p < .001 versus control. Data are presented as the mean ± SEM. Data in (A) were generated from the GSE47460 dataset and data in (B) were generated from the GSE76808 dataset

We analyzed the public datasets GSE47460 (IPF = 122, control = 92) and GSE76808 (SSc‐PF = 14, control = 4), and found a decreased LDLR expression in the lungs of both IPF and SSc‐PF patients compared to controls (Figure 1A and B). We then demonstrated a decreased *LDLR* mRNA level in the lungs of 24 IPF patients compared to that of 15 controls (Figure 1C). *LDLR* mRNA levels were negatively correlated with *COL3A1*, *ACTA2*, *CXCL13* and *CASP3* mRNA expression in the IPF lungs (Supporting information Figure S1). Western blot further showed that LDLR protein levels declined in SSc‐PF and IPF lungs compared with controls (Figure 1D). Immunofluorescence double staining assay showed that LDLRs were predominantly expressed in ATII and fibroblast cells, but slightly expressed in endothelium. Moreover, LDLR levels were significantly decreased in ATII and fibroblast cells, while they were increased in endothelium of SSc‐PF and IPF lungs compared with controls (Figure 1E, 1E1 and Supporting information Figure S2). The aberrant expression of LDLR was further confirmed in FAC‐sorted ATII, endothelial and fibroblast cells of the tissues (Figure 1F). The gating strategy for the isolation of ATII, endothelial and fibroblast cells from human lungs is shown in Supporting information Figure S3.

**HIGHLIGHTS**

- Disrupted LDL–LDLR metabolism in PF patients and mice.
- LDLR deficiency induces fibrosis.
- Atorvastatin combines PCSK9 antibody blunted pulmonary fibrosis.

To find which lipid parameter is regulated by LDLR reduction and associated with PF, the levels of LDL, high‐density lipoprotein (HDL) and total cholesterol (TC) and triglyceride (TG) levels were analyzed in the plasma of 185 SSc‐PF patients, 55 IPF patients and 1642 controls. Compared with the controls, the SSc‐PF and IPF patients showed significantly high LDL levels and a high LDL/HDL ratio, but low HDL levels (Figure 1G–I). Other lipids, including TC and TG, did not show any significant differences (Figure 1J and K). Additionally, LDL levels and the LDL/HDL ratio were increased in PF (IPF > SSc‐PF > SSc > controls). The detailed clinical information is presented in Supporting information Table S1.

Taken together, these results indicate that disturbed LDL–LDLR axis is a common mechanism in ILD diseases, and may be associated with PF involvement.

### 2.2 Aberrant LDLR and increased LDL in BLM‐induced mouse PF

**FIGURE 2:**
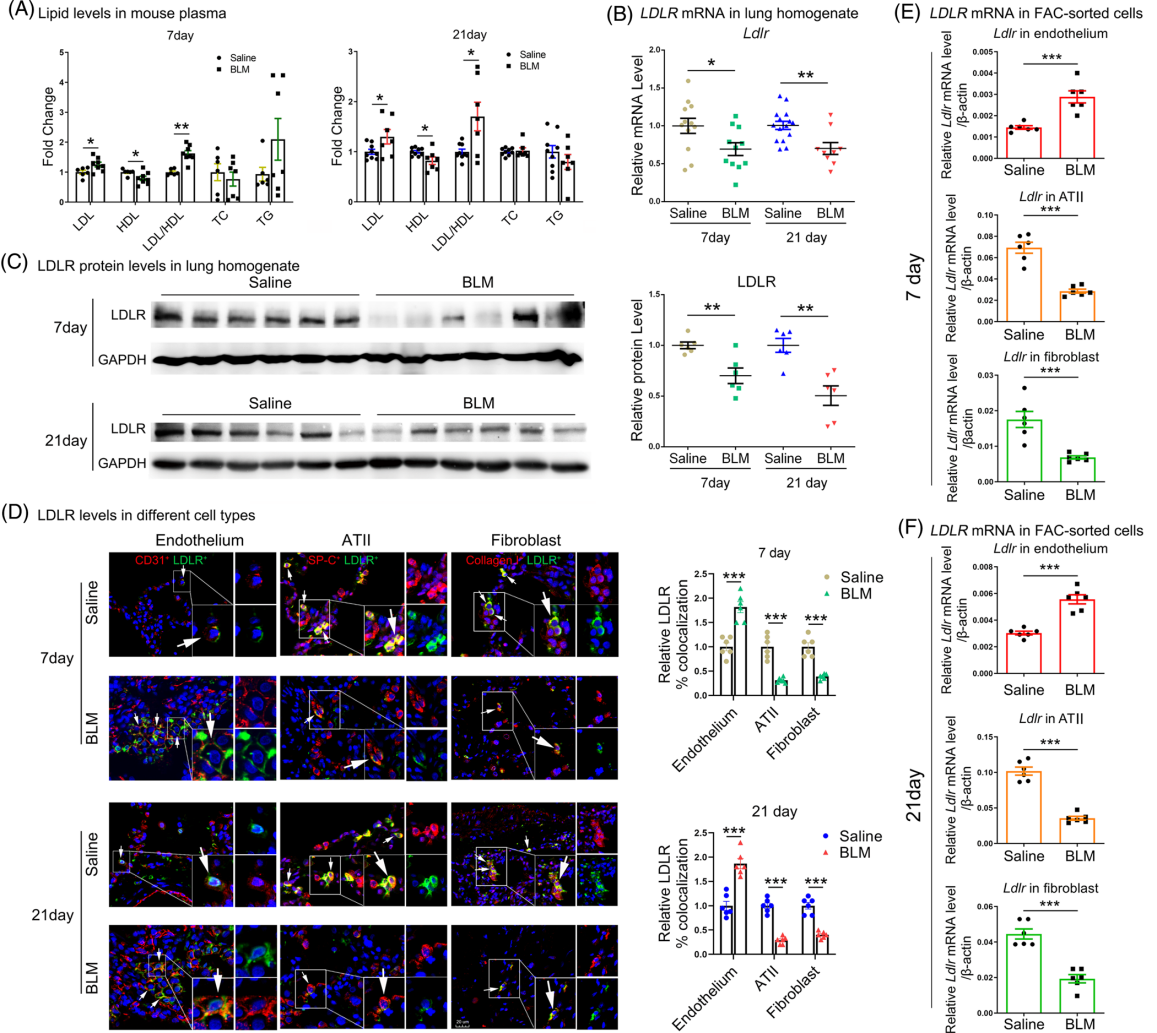
Dysregulated LDLR and increased LDL levels in BLM‐induced mouse PF. (A) Lipid levels in the plasma of BLM‐induced SSc‐PF mouse at day 7 and 21. (B–C) Detection of LDLR mRNA and protein expression in the lungs from BLM‐ or saline‐treated WT mice at day 7 and 21 by qPCR and western blot analysis. (D) Immunofluorescence analysis of LDLR protein levels in endothelial, ATII and fibroblast cells. (E–F) *Ldlr* levels in FAC‐sorted ATII and fibroblast cells from BLM‐ or saline‐treated WT mice at day 7 and 21 by qPCR analysis. Scale bars: 20 μm. *N* ≥ 6 per group. *p < .05, **p < .01, ***p < .001 versus control

Subsequently, LDL–LDLR metabolism was analyzed in mice with PF induced by intratracheal instillation of 2.5 mg/kg BLM. As shown in Figure 2A, LDL levels and the LDL/HDL ratio were increased in both the early fibrotic and the advanced fibrotic stages. In addition, both the mRNA and protein expression of LDLR were significantly reduced in the lung tissues of PF mouse, as shown by qPCR and western blot analysis (Figure 2B and C). Immunohistofluorescence analysis further showed that LDLR was downregulated in lung ATII and fibroblast cells, while it was upregulated in endothelial cells on both days 7 and 21 after BLM instillation, compared to saline‐treated control mice (Figure 2D and Supporting information Figure S4). Furthermore, the dysregulation of LDLR in ATII, endothelial and fibroblast cells was confirmed in FAC‐sorted cells (Figure 2E and F). These results are consistent with the changes observed in SSc‐PF and IPF patients. Besides, disrupted LDL–LDLR expression was also observed in other three mouse PF models, including BLM‐induced SSc‐PF, DNA Topoisomerase I, Complete Freund's Adjuvant (TopoI‐CFA)‐induced PF and graft‐versus‐host disease (GVHD)‐induced PF (Supporting information Figure S5). These data suggest that LDL–LDLR expression was persistently disrupted in different types of mouse PF.

### 2.3 *Ldlr* knockout accelerated and exacerbated BLM‐induced PF in mice

**FIGURE 3:**
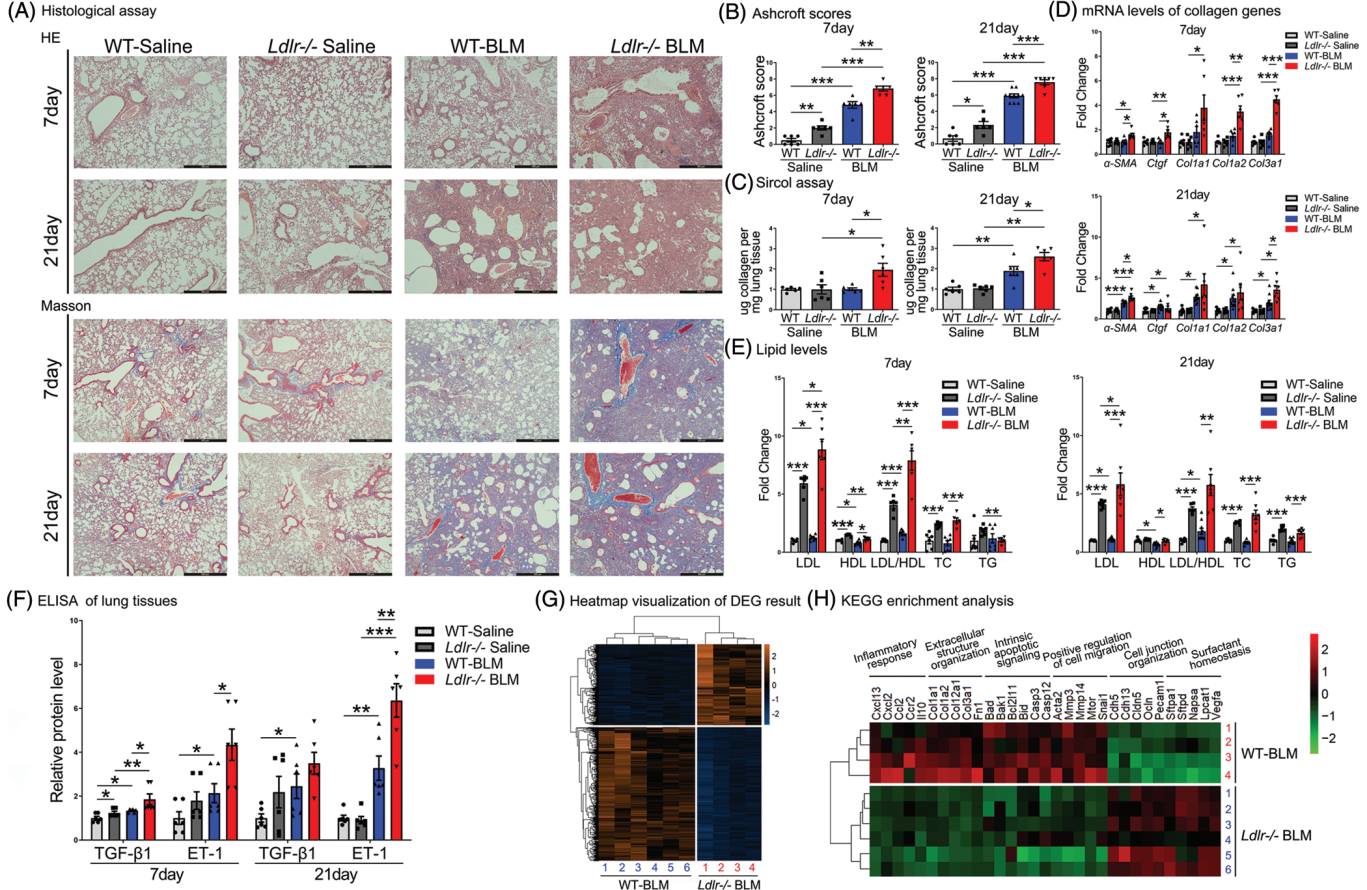
*Ldlr* knockout exacerbated BLM‐induced PF. (A–C) Pulmonary tissue sections were stained with H&E and Masson's trichrome, and the Ashcroft score was calculated. Scale bar: 500 μm. (C) Soluble collagen synthesis in lung homogenate. (D) qRT‐PCR analysis of fibrosis‐related genes in mouse lungs. (E) Plasma lipid levels. (F) Measurement of TGF‐β1 and ET‐1 levels at day 7 and 21 after BLM treatment. (G) Heatmap of all of the differentially expressed genes in *Ldlr−*/− mice and WT mice in response to BLM administration. (H) The expression profiles of inflammation‐, ECM‐, apoptosis‐, migration‐, junction‐ and surfactant homeostasis‐related genes. mRNA levels, cell counts and lipid levels were normalized to the saline‐treated group. *N *= 6–10 per group in data (A) to (F) and *N *= 4–6 per group in data (G) and (H). *p < .05, **p < .01, ***p < .001 versus control. Data are presented as the mean ± SEM

To explore the role of LDL–LDLR in PF, saline, or BLM (2 mg/kg) was intratracheally instilled into *Ldlr−*/− and wild‐type (WT) mice. H&E (Haematoxylin, eosin), Masson's trichrome staining and Ashcroft score assay revealed that BLM induced a significant increase in the injured and fibrotic areas in *Ldlr−*/− mice compared with WT mice, on both days 7 and 21 (Figure 3A and B). Sircol and qPCR assay further revealed that soluble collagen content and mRNA levels of fibrotic‐related genes, including *Col1a1*, *Col1a2*, *Col3a1*, *α‐Sma* and *Ctgf*, significantly increased in *Ldlr−*/− mice compared with WT mice, in response to BLM treatment on both days 7 and 21 (Figure 3C and D). It is worth noting that BLM induced a fibrotic change in *Ldlr−*/− mice, rather than in WT mice, as early as day 7, suggesting that mice with *Ldlr* deficiency are more susceptible to BLM‐induced PF. We next found that the relative amounts of LDL and the ratio of LDL/HDL significantly increased in *Ldlr−*/− mice on days 7 and 21 (Figure 3E). All these histopathological changes were associated with lower survival rate after BLM instillation in *Ldlr−*/− mice (6 of 10 mice alive at 7 days and 6 of 10 at 21 days in *Ldlr−*/− mice versus 8 of 10 mice at 7 days and 10 of 10 mice at 21 days in WT mice). Enzyme‐linked immunosorbent assay (ELISA) of the lung tissues showed that ET‐1 and TGF‐β1 protein levels increased in *Ldlr−*/− mice compared with WT mice at baseline, and that these levels were further augmented in BLM‐treated *Ldlr−*/− mice on days 7 and 21 (Figure 3F). Additionally, cell counts of BALF (bronchoalveolar lavage fluid) revealed that leukocytes and lymphocytes were significantly elevated in *Ldlr−*/− mice compared with WT mice (Supporting information Figure S6A). Consistently, the mRNA levels of *IL‐6*, *IL‐10*, *Ccl2*, *Ccl12* and *Cxcl13* (inflammatory cytokines) and *Ccr2*, *Cx3cr1* and *Lcp2* (cytokine receptors) were higher in *Ldlr−*/− mouse lungs. In contrast, the levels of *Flt3l*, whose deficiency worsens lung fibrosis,^25^ were lower in *Ldlr−*/− mouse lungs (Supporting information Figure S6B). To further explore whether severe fibrosis observed in *Ldlr−*/− mice is just a result of exacerbated inflammation, a TGF‐β1‐induced PF model was constructed by intratracheally instilling 5 × 10^8^ plaque‐forming units (PFU) adenovirus encoding active transforming growth factor beta‐1 (AdTGF‐β1) into *Ldlr−*/− and WT mice. As expected, AdTGF‐β1‐induced a significant increase in the fibrotic areas in *Ldlr−*/− mice compared with WT mice on day 21, as measured by HE, Masson, Ashcroft scores and Sircol assay. Therefore, we conclude that *Ldlr−*/− deletion also exacerbates the fibrosis process, not merely inflammation (Supporting information Figure S7). To further investigate the molecular events underlying the role of LDLR deficiency in PF, a quantitative assessment of transcriptome among *Ldlr−*/− BLM and WT‐BLM group was performed (Figure 3G). Enrichment analysis showed the genes involved in intrinsic apoptotic signalling, cell migration, inflammatory response and ECM (extracellular matrix) were significantly upregulated, whereas genes involved in cell junctions and surfactant homeostasis were downregulated in BLM‐treated *Ldlr−*/− mice compared with BLM‐treated WT mice (Figure 3H). The transcriptome differences between WT‐Saline and *Ldlr−*/− Saline were performed, and found that the inflammation‐related genes (such as *Ccl2/ Ccl7/ Ccl8/ Ccl12/Cxcl15/Cxcl13/Cxcl1*…) were higher in *Ldlr−*/− mice as well. Additionally, genes involved in apoptotic process were also enriched in *Ldlr−*/− mice (Supporting information Figure S8).

### 2.4 Increased apoptosis of endothelial and epithelial cells in *Ldlr−*/− mouse lungs

**FIGURE 4:**
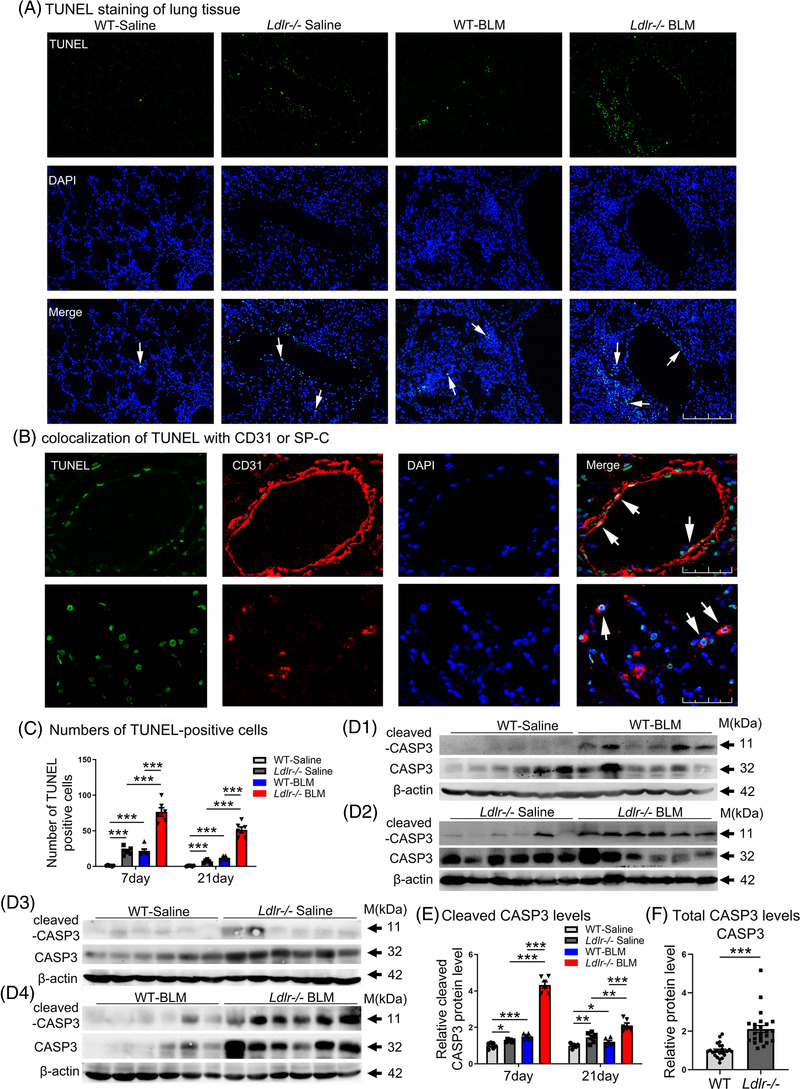
Increased apoptosis in lungs of BLM‐treated *Ldlr−*/− mice. (A) Representative TUNEL staining images of lung sections at day 7. Scale bar: 250 μm. (B) Co‐localization of positive TUNEL labelling with anti‐CD31 or anti‐SP‐C staining from BLM‐treated *Ldlr−*/− mice at day 7. Scale bar: 25 μm. (C) Numbers of apoptotic cells at day 7. (D) Western blot analysis of cleaved caspase‐3 and total caspase‐3 levels in the lungs at day 7. D1: caspase 3 activation in WT mouse lungs; D2: caspase 3 activation in *Ldlr−*/− mouse lungs; D3: The endogenous levels of cleaved and total caspase 3 in WT and *Ldlr−*/− mice without BLM treatment. D4: The levels of cleaved and total caspase 3 in WT and *Ldlr−*/− mice with BLM treatment. (E−F) Densitometric values of cleaved caspase‐3 (E) and total caspase‐3 (F) at both days 7 and 21 in the lungs. *N* ≥ 6 per group in (A to E). *N* = 24 per group in (F). *p < .05, **p < .01, ***p < .001 versus control. Data are presented as the mean ± SEM

As shown in Figure 4A and C, and Supporting infromation Figure S9A, TUNEL‐positive cells dramatically increased in *Ldlr−*/− mice compared to WT mice after BLM treatment. Furthermore, the co‐localization of TUNEL‐positive cells with CD31‐ and SP‐C‐positive cells indicated that TUNEL‐positive cells are indeed endothelial and ATII cells (Figure 4B). Western blot analysis of lung homogenates revealed that BLM effectively induced cleaved caspase‐3 production in *Ldlr−*/− mice and WT mice (Figure 4D1–D2 and Supporting information Figure 9B1–B2). Seven days after BLM treatment, significantly higher cleaved caspase‐3 levels were observed in *Ldlr−*/− mice compared with WT mice (Figure 4D3, 4D4 and 4E). On day 21, a similar increase was observed (Supporting information Figure S9B3–B4 and Figure 4E). Importantly, we revealed that *Ldlr−*/− mice exhibit increased total caspase‐3 expression, which may explain the susceptibility of total caspase‐3 to cleavage and then their cells to apoptosis (Figure 4D3–4 and F, and Supporting information Figures S9B3–4).

We also measured the expression levels of ROS clearing genes, which play a central role in protecting against intrinsic apoptosis,^26^ and found that superoxide dismutases and catalase were significantly reduced in *Ldlr−*/− lungs compared with WT lungs (Supporting information Figure S9C).

### 2.5 *Ldlr* deletion increased fibroblast‐like endothelial and ATII cells in vivo

**FIGURE 5:**
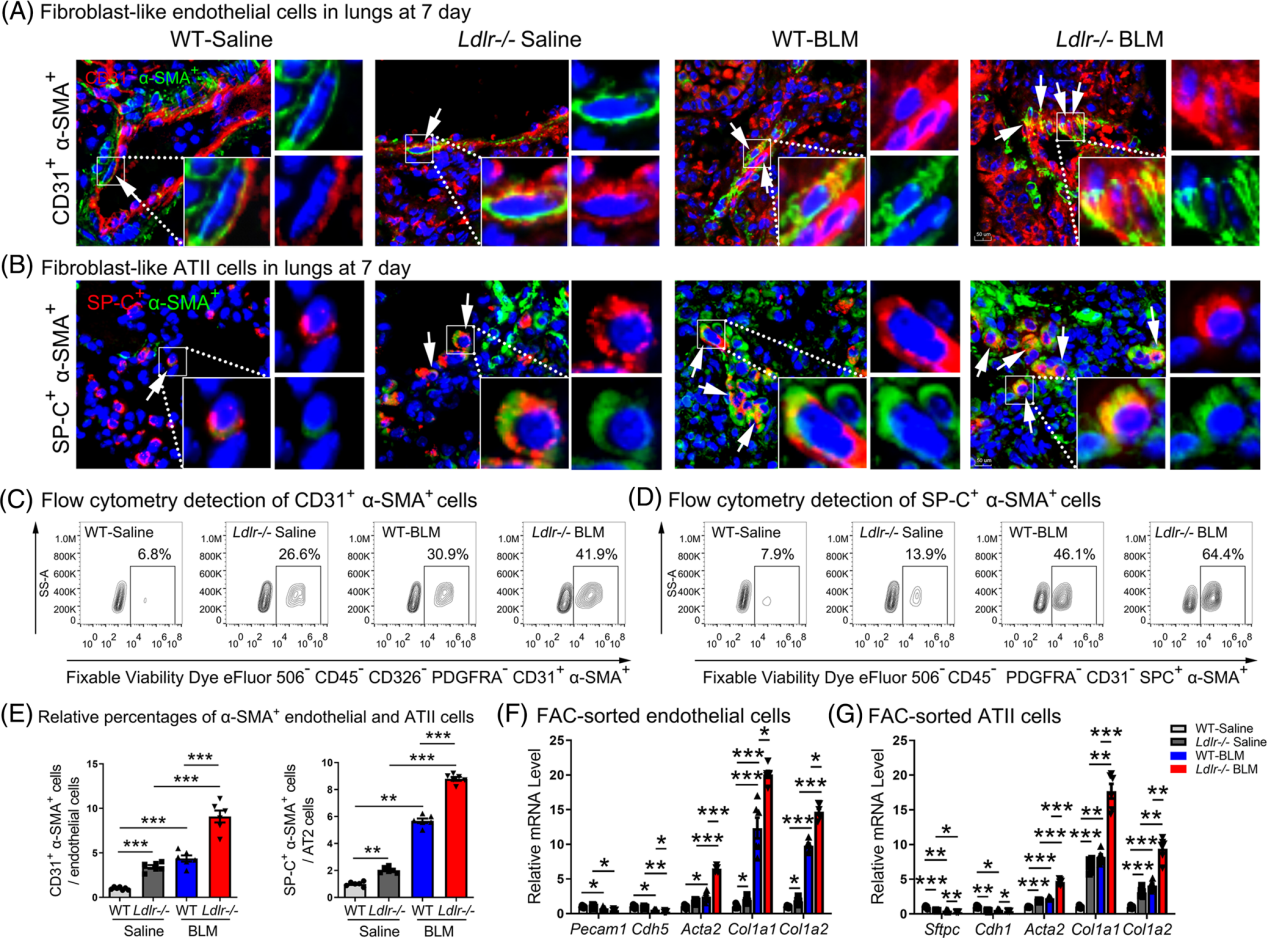
Fibroblast‐like endothelial and ATII cells were induced in *Ldlr−*/− mouse lungs. (A) Immunofluorescence of the myofibroblast marker α‐SMA (green) and the endothelial marker CD31 (red) in the lungs. Scale bar: 50 μm. (B) Immunofluorescence of the myofibroblast marker α‐SMA (green) and the ATII marker SP‐C (red) in the lungs. Scale bar: 50 μm. (C–E) Counting of Fixable Viability Dye eFlour 506^–^ CD45^–^ CD326^–^ PDGFRA^–^ CD31^+^ α‐SMA^+^ (C and left panel of E) and Fixable Viability Dye eFlour 506^–^ CD45^–^ PDGFRA^–^ CD31^–^ SP‐C^+^ α‐SMA^+^ cells in mice (D and right panel of E). (F–G) mRNA levels of endothelial, epithelial and mesenchymal markers in sorted endothelial and epithelial cells, as analyzed by qPCR. mRNA levels were normalized to the saline‐treated group. *N* ≥ 6 per group. *p < .05, **p < .01, ***p < .001 versus control. Data are presented as the mean ± SEM

Besides apoptosis, the post‐injury cells undergo other pathological changes upon the inflammatory and fibrotic challenges.^7^, ^27^ We observed co‐localization of α‐SMA in CD31‐expressing endothelium and SP‐C‐expressing epithelium within fibrotic foci in *Ldlr−*/− mice, which was strongly augmented in BLM‐treated *Ldlr−*/− mice at day 7. The number of CD31^+^α‐SMA^+^ and SP‐C^+^α‐SMA^+^ cells was increased in the *Ldlr−*/− BLM group compared with the WT BLM group (Figure 5A and B). Flow cytometry further demonstrated that the percentages of α‐SMA positive cells were both increased in living endothelial (Fixable Viability Dye eFlour 506^–^ CD45^–^ CD326^–^ PDGFRA^–^ CD31^+^ α‐SMA^+^) and ATII (Fixable Viability Dye eFlour 506^–^ CD45^–^ PDGFRA^–^ CD31^–^ SP‐C^+^ α‐SMA^+^) cells of the *Ldlr−*/− group compared with the WT group (Figure 5C–E). The gating strategy for indentation of α‐SMA‐positive cells in endothelial and type II alveolar cells from the lungs of mouse is shown in Supporting information Figure S10A–E. At 21 days, BLM‐treated *Ldlr−*/− mice still exhibited more fibroblast‐like endothelial and ATII cells, and the number of fibroblast‐like cells was more than that in the WT BLM group, indicating the severity of fibrosis in *Ldlr−*/− mice without any remission (Supporting information Figure S11A).

Correspondingly, primary endothelial (Fixable Viability Dye eFlour 506^–^ CD45^–^ CD326^–^ PDGFRa^–^ CD31^+^) and ATII (Fixable Viability Dye eFlour 506^–^ CD45^–^ CD31^–^ CD326^+^ PDGFRa^–^ Ter‐119^–^ CD104^–^) cells sorted from *Ldlr−*/− mice also showed reduced mRNA levels of the endothelial markers *Pecam1* and *Cdh5* and the ATII markers *Cdh1* and *Sftpc*, while mRNA levels of the mesenchymal markers *Acta2, Col1a1* and *Col1a2* (Figure 5F and G) were increased. Additionally, similar changes in these markers were observed in lung homogenate (Figure 3D, Supporting information Figure S11B). Our results strongly support our hypothesis that *Ldlr−*/− deletion not only drives the induction of apoptosis, but also induced the accumulation of fibroblast‐like endothelial and ATII cells in vivo.

### 2.6 Disrupted LDL–LDLR metabolism induced apoptosis, accumulation of fibroblast‐like cells and fibrosis in vitro

**FIGURE 6:**
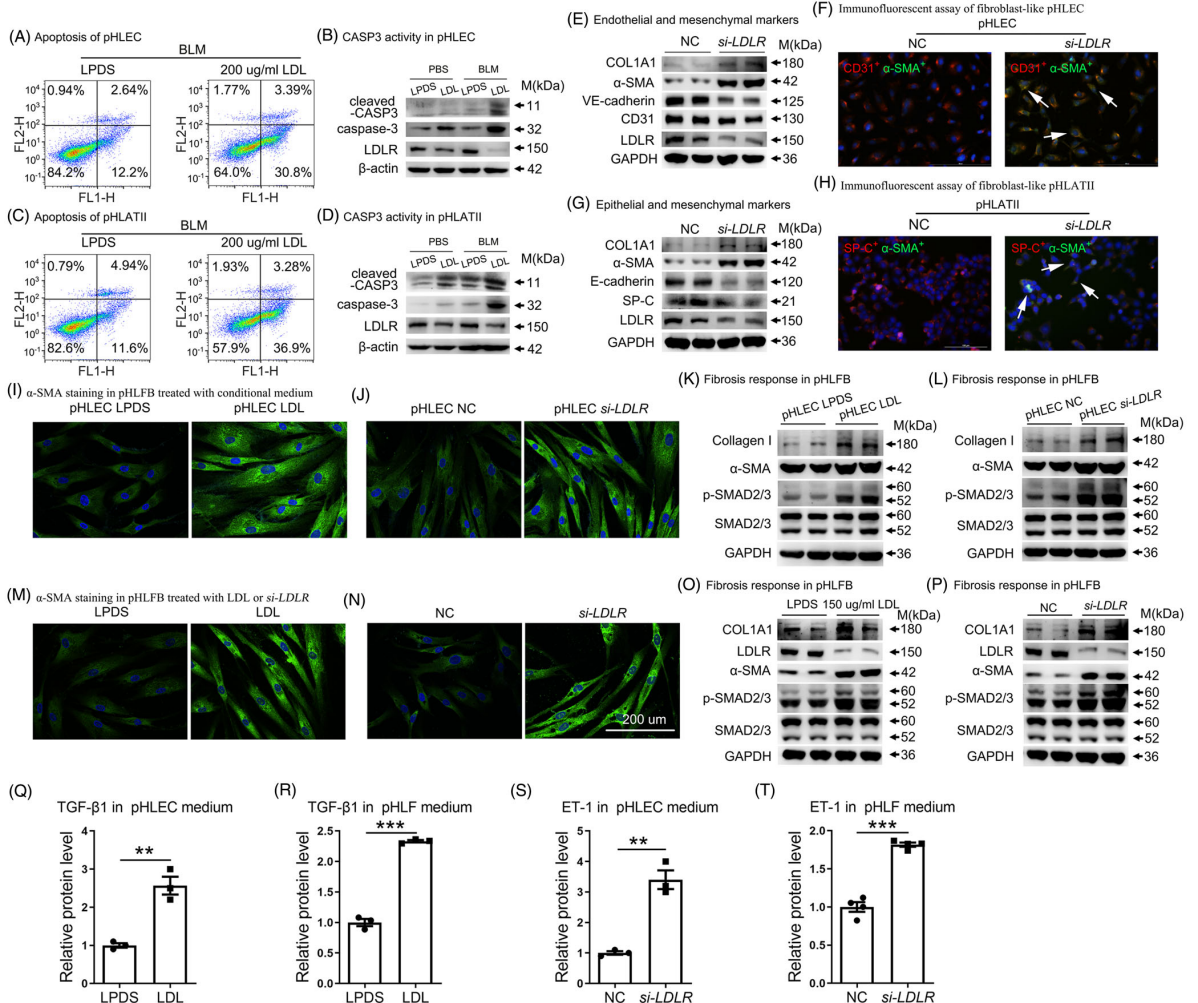
LDL and *LDLR* knockdown induced apoptosis, fibroblast‐like endothelial and ATII cells and fibrosis. Examination of apoptosis in pHLEC cells (A–B) and pHLATII cells (C–D) after LDL stimulation by flow cytometry and western blot. (E–F) Effects of *si‐LDLR* on the induction of fibroblast‐like endothelial cells based on western blot and immunofluorescent assay. (G–H) Effects of *si‐LDLR* on the induction of fibroblast‐like epithelial cells based on western blot and immunofluorescent assay. (I–J) Immunofluorescence of α‐SMA in PHLF after incubation with conditioned medium from LDL‐ or *si*‐*LDLR*‐treated endothelial cells. (K–L) Collagen, α‐SMA and p‐SMAD2/3 levels, as analyzed by western blot. (M–N) Immunofluorescence and western blot analyses were performed on cells treated with LDL or *si‐LDLR* for 12 and 48 h, respectively. (O–P) Collagen, α‐SMA and p‐SMAD2/3 levels, as analyzed by western blot. (Q–R) ELISA analysis of TGF‐β1 in culture medium from LDL‐treated pHLEC and PHFL cells, respectively. (S–T) ELISA analysis of ET‐1 levels in the culture medium from LDLR‐deficient pHLEC and PHFL cells. Lipoprotein deficient serum (LPDS) and control siRNA were used as controls. Scale bar: 200 μm. *p < .05, **p < .01, ***p < .001 versus control. Data are presented as the mean ± SEM of three independent experiments

To test whether LDL causes apoptosis in endothelial and ATII cells, primary human lung endothelial cells (pHLEC) and primary human lung ATII cells (pHLATII) were incubated with 200 μg/mL LDL in the presence of PBS or 50 μg/mL BLM. Results revealed LDL alone exhibited slight effect on cell apoptosis (Supporting information Figure S12), but enhanced BLM‐induced apoptosis and cleaved caspase‐3 expression (Figure 6A–D and Supporting information Figure S13A–D).

Considering fibroblast‐like endothelial and ATII cells were increased in *Ldlr−*/− mice, we determined whether these fibroblast‐like changes were induced by LDLR reduction. Very significant changes were detected in LDLR‐deficient pHLEC, including loss of endothelial markers CD31 and VE‐cadherin, but there was a gain of the mesenchymal markers α‐SMA and collagen I. Immunofluorescence staining indicated enhanced expression of the myofibroblast marker α‐SMA in CD31 expressing pHLEC, further supporting the induction of fibroblast‐like endothelial cells by *LDLR* knockdown (Figure 6E and F and Supporting information Figure S13E and F). Correspondingly, fibroblast‐like ATII was also induced in LDLR‐deficient pHLATII, as indicated by epithelial and mesenchymal marker detection and coimmunofluorescence analysis (Figure 6G and H and Supporting information Figure S13G and H). Primary human lung fibroblasts (pHLF) were treated with conditioned medium from LDL‐ or *si‐LDLR*‐treated pHLEC cells, which promoted fibrotic responses, as shown by immunofluorescence and Western blot analysis of α‐SMA, p‐SMAD2/3 and collagen levels (Figure 6I–L and Supporting information Figure S13I‐J and K‐L). Similarly, fibrotic responses were induced in pHLF by conditioned medium from LDL‐ or *si‐LDLR*‐treated pHLATII cells (Supporting information Figure S13M–N). Moreover, LDL‐ and *si‐LDLR*‐treated fibroblasts showed the same changes as those observed in conditioned medium‐treated cells (Figure 6M–P, and Supporting information Figure S13O–R). We next explored the underlying mechanisms and found that LDL induced TGF‐β1 secretion (Figure 6Q–R, and Supporting information Figure S13S), while LDLR deficiency induced ET‐1 secretion in endothelial, epithelial and fibroblast cells (Figure 6S–T, and Supporting information Figure S13T).

### 2.7 Atorvastatin combined with alirocumab alleviated BLM‐induced PF in mice

**FIGURE 7:**
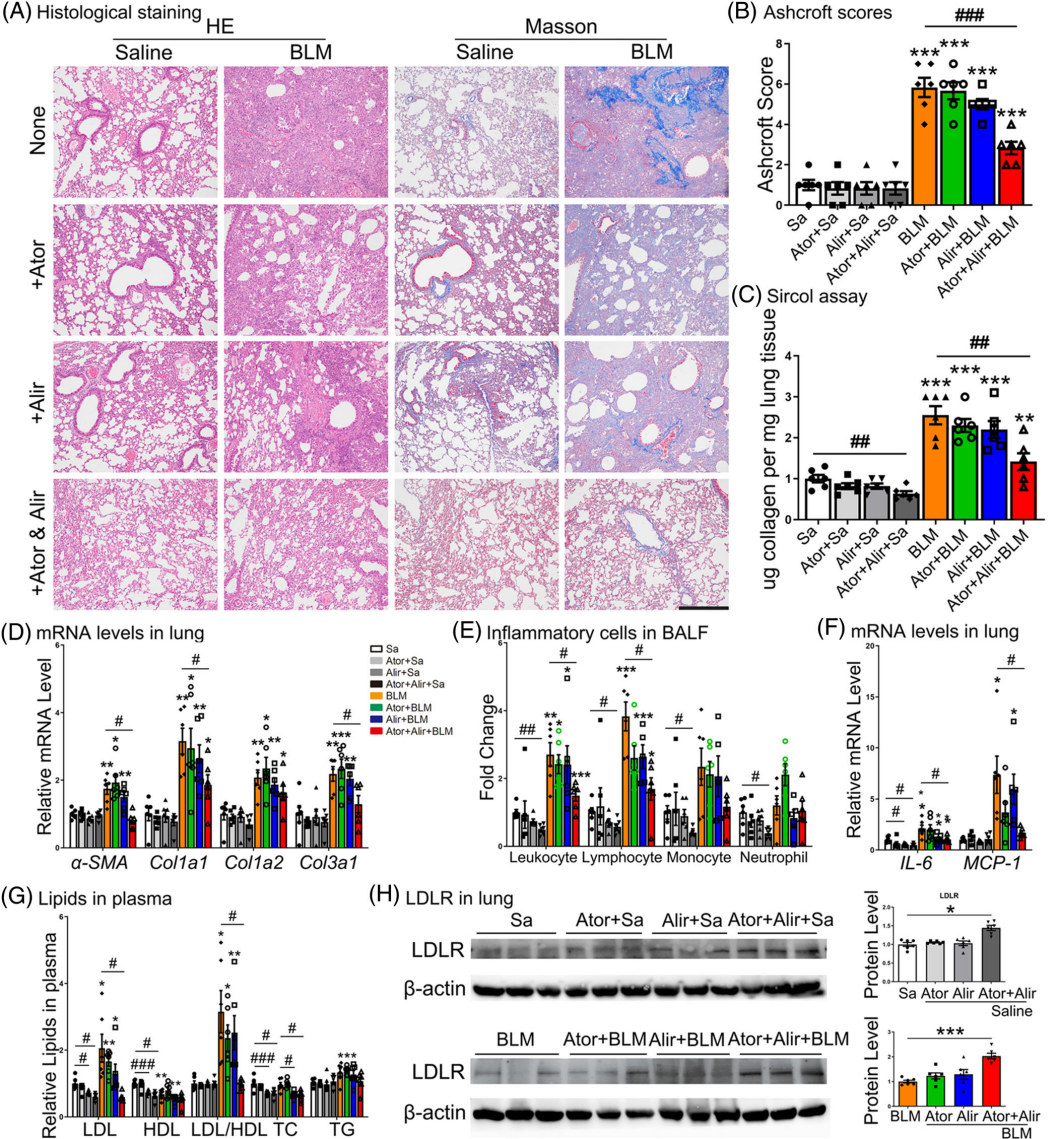
Atorvastatin combined with alirocumab released BLM‐induced PF in vivo. (A–B) Lung tissue sections were stained with H&E and Masson's trichrome and the Ashcroft score was calculated. Scale bar: 500 μm. (C–D) Measurements of ECM content and the expression of collagen genes in lungs of mice receiving different treatments by Sircol assay and qRT‐PCR analysis, respectively. (E) Cell counts in BALF at baseline and 21 days after BLM treatment. (F) mRNA levels of *IL‐6* and *MCP‐1* in mouse lungs. (G) Plasma lipid levels. (H) LDLR expression in lungs of mice after treatment with atorvastatin, alirocumab or both in the saline group and the BLM group. *N* ≥ 6 per group. *p < .05, **p < .01, ***p < .001 versus saline control; #*p* < .05, ##*p* < .01, ###*p* < .001 within different treatment groups

We found that PCSK9 levels were elevated in the plasma of SSc‐PF and IPF patients, and BLM‐induced PF mice, compared with controls (Supporting information Figure S14A–C). Next, mice were treated with atorvastatin and/or alirocumab before and after treatment with a higher dose of BLM (4 mg/kg). On day 21, lungs from mice treated with the combination of atorvastatin and alirocumab showed decreased lesion sizes and fibrotic areas, while only a slight decrease was observed in the atorvastatin group and the alirocumab group compared with BLM‐treated control mice, as demonstrated by histological staining, Ashcroft score, Sircol assay and fibrotic genes analysis (Figure 7A–D).

Inflammatory cells in BALF, including leukocytes and lymphocytes, also decreased after combination treatment (Figure 7E). Histopathological and molecular changes were accompanied by lower mortality in the combined treatment group than in the BLM‐treated control group (3 of 10 mice died, weight 22.6 ± 1.5 g in combination treatment group versus 9 of 15 mice died, weight 17.0 ± 2.1 g in BLM without any treatment). In addition, combined treatment inhibited the BLM‐induced elevation of LDL levels and the LDL/HDL ratio, returning them to physiological levels (Figure 7F). We next found that combination treatment significantly enhanced LDLR protein expression, while atorvastatin or alirocumab alone only caused a slight increase (Figure 7G). However, combined treatment failed to prevent the PF progression in *Ldlr−*/− mice, supported by more severe lung injury and fibrosis than that in WT mice (Supporting information Figure S15A–C). To confirm a true anti‐fibrotic effect of our combination treatments, new experiments were designed. Atorvastatin (orally, 5 mg/kg/d) and alirocumab (subcutaneous injection, 3 mg/kg/week) were delivered to mice from day 7 to 21 after BLM treatment. Results showed combination treatment showed an inhibitory effect on PF progression as measured by HE, Masson and collagen‐related gene levels (Supporting information Figure S16).

### 2.8 Atorvastatin combined with alirocumab reversed apoptosis, fibroblast‐like changes of endothelial and ATII cells and BLM‐induced pathway imbalance

**FIGURE 8:**
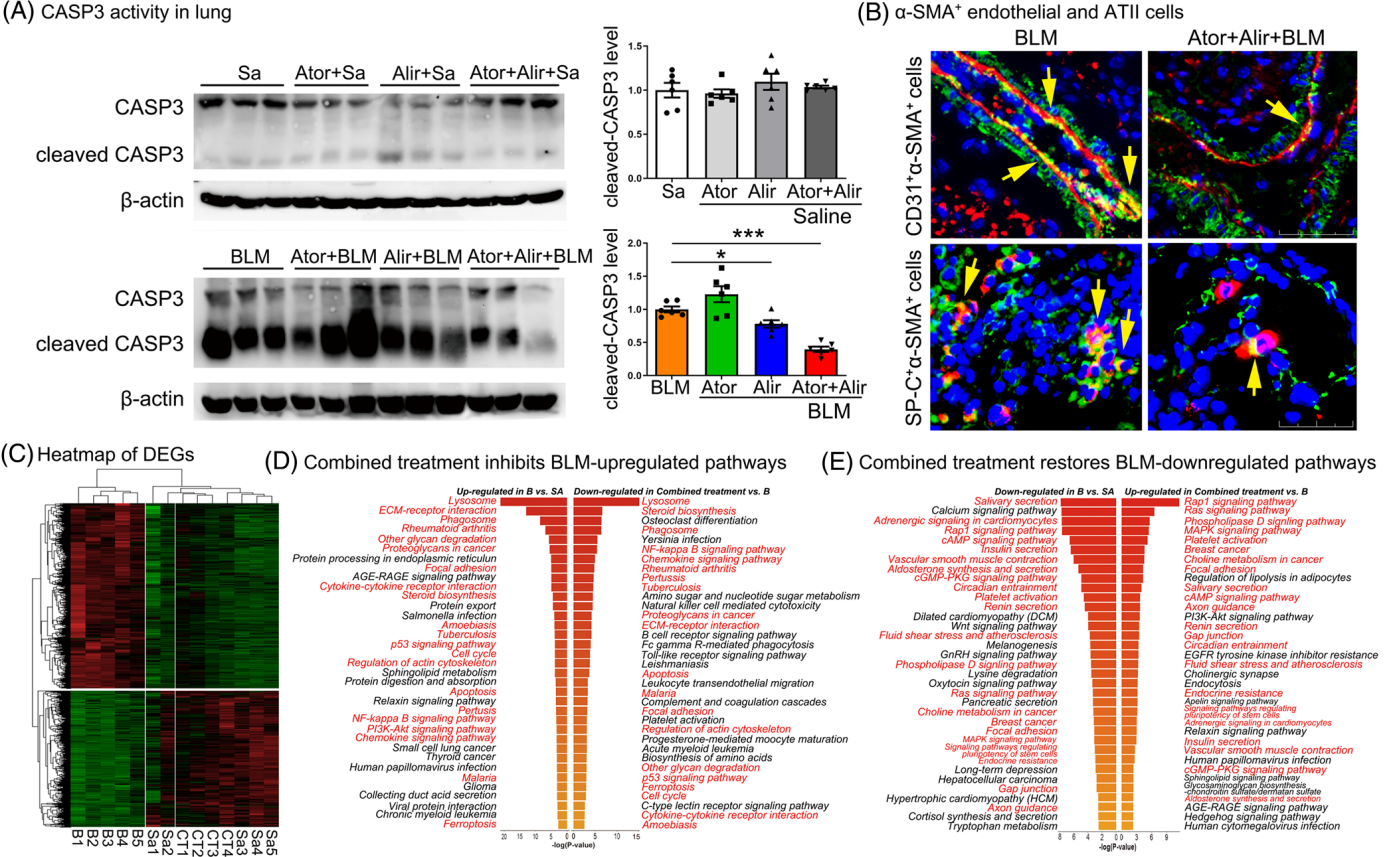
Atorvastatin combined with alirocumab inhibited BLM‐induced EndoMT, EMT and pathway imbalance in mice. (A) Western blot analysis of cleaved caspase‐3 and total caspase‐3 levels in the lungs. (B) Immunofluorescence of CD31^+^α‐SMA^+^ cells and SP‐C^+^α‐SMA^+^ cells in the combined treatment group and the BLM control group. Scale bar: 50 μm. (C–E) Heatmap and KEGG analysis of the differentially expressed genes. Sa, Saline; B, BLM; CT, combined treatment. *N* ≥ 6 per group in data (A) to (B) and *N* ≥ 4 per group in data (C) to (E). *p < .05, **p < .01, ***p < .001 versus saline control; #*p* < .05, ##*p* < .01, ###*p* < .001 within different treatment groups

Next, we found that combination treatments prominently suppressed cleaved caspase‐3 in the lungs of mice (Figure 8A). Moreover, immunofluorescent assays revealed that the numbers of CD31^+^α‐SMA^+^ double‐positive and SP‐C^+^α‐SMA^+^ double‐ positive cells were both decreased, suggesting reduced fibroblast‐like endothelial and ATII cells in the combination group (Figures 8B and Supporting information Figure S17A–B). Consistently, mRNA levels of the endothelial markers *CD31* and *Cdh5* and the ATII marker *Sftps* were increased, while mRNA levels of the mesenchymal markers *S100a4* and *Fn1* were reduced (Supporting information Figure S17C). To further characterize the molecular mechanisms underlying the protective effects of combined treatment in the mouse PF model, differentially expressed mRNAs were sequenced. As shown in the heatmap in Figure 8C, the gene expression patterns in mice from the combined treatment groups were similar to those in mice from the saline control group. KEGG analysis further revealed the upregulated pathways in the BLM group, which are mainly involved with ECM–receptor interaction, cytokine–cytokine receptor interaction, apoptosis and NF‐κB signalling. The mRNAs were significantly restored to normal levels after combined treatment (Figure 8D). Moreover, pathways that were downregulated in the BLM group, which were mainly involved in salivary secretion, Rap1 signalling, camp signalling and gap junctions, were also almost restored to physiological levels after combined treatment (Figure 8E).

## 3 DISCUSSION

PF is an irreversible lung disease with a poor prognosis.^28^ In the current study, we provide several lines of evidence suggesting that the abnormal LDL–LDLR metabolism plays an important role in PF. This finding provides new insight into PF and suggests new targets for therapy. This is the first report to show that LDL significantly increased in SSc‐PF and IPF patients in a large cohort. We further confirmed epithelial cells and fibroblasts are predominant LDLR‐expressing cell types, which are responsible for the reduction in pulmonary LDLR levels in PF. The dysregulation of LDLR in endothelial, ATII and fibroblast cells was in agreement with the previous reports of single‐cell RNA‐sequencing data, or FAC‐sorted or culture primary cells.^1^, ^29^, ^30^ Subsequently, similar LDL–LDLR changes were obtained in different types of PF mice. These clinical and experimental results supported the notion that abnormal LDL–LDLR metabolism is a common event in PF.

We then confirmed that *Ldlr* depletion led to the exacerbation of PF in mice as early as 7 days after BLM exposure. Our transcriptome data not only reveal the upregulation of intrinsic apoptotic pathways in *Ldlr−*/− mice lungs, but also uncover the activation of cell migration‐related pathways. We observed increased caspase‐3 activity in *Ldlr−*/− mice, and apoptosis of both endothelial and epithelial cells. In addition, we observed more fibroblast‐like endothelial and ATII cells in *Ldlr−*/− than in WT mice. Our ELISA results showed both TGF‐β1 and ET‐1, which were induced by LDL and *si‐LDLR*, respectively, were significantly increased in *Ldlr−*/− mice compared to WT mice. Multiple studies have found that TGF‐β1 and ET‐1 can induce PF by promoting fibroblast differentiation and collagen deposition.^31^, ^32^, ^33^, ^34^, ^35^ Therefore, it seems reasonable that *Ldlr−*/− mice are more susceptible to BLM or TGF‐β1‐induced PF. In vitro experiments further clarified that apoptosis, and accumulation of fibroblast‐like endothelial and ATII cells were induced by LDL and LDLR deficiency, respectively. Thus, TGF‐β1 and ET‐1 are potential contributors to LDL‐induced apoptosis and LDLR deficiency‐induced fibroblast‐like changes. Although LDL and *si‐LDLR* induced different biological responses in cells, they had a common effect on fibrogenesis. Therefore, it seems reasonable to speculate that increased apoptosis and fibroblast‐like cells are involved in the profibrotic effects of perturbed LDL–LDLR metabolism. Moreover, fibroblasts directly treated with LDL or *si‐LDLR* induced similar fibrotic responses as well. Though LDLR was increased in endothelial cells of PF patients and mice, the in vitro and in vivo experiments consistently demonstrated the protective effect of LDLR in endothelial cell function. Overall, our findings have important clinical relevance; clinicians should pay careful attention to LDL and LDLR levels in patients with PF or other pulmonary defects.

Atorvastatin and alirocumab are safe and widely used LDL‐lowering drugs by improving LDLR expression. Initial results showed that atorvastatin or alirocumab alone had little or no effects. However, the combined treatment led to significant reductions in several proinjury and profibrotic markers including collagens and inflammatory cells. Notably, apoptosis, accumulation of fibroblast‐like endothelial and ATII cells, which were all amplified in LDLR‐deficient mice and cells, were significantly suppressed by combined treatment. Correspondingly, reduced levels of LDL were observed in mice receiving combined treatment, suggesting that the cure of lung fibrosis involves LDL metabolism. Indeed, neither atorvastatin alone nor alirocumab alone could effectively block BLM‐induced increases in LDL levels and the LDL/HDL ratio, indicating that either medication alone is insufficient. We detected the effect of statin on isolated endothelial, ATII and fibroblast cells, and found that atorvastatin slightly upregulated LDLR protein expression in endothelial, ATII and fibroblast cells. Besides, atorvastatin exhibited obvious cytotoxicity when the dosage was higher than 5 μM in these cells. However, we found no changes in cleaved CASP3 expression after atorvastatin (5 μM) treatment, suggesting atorvastatin did not affect cell apoptosis. Besides, atorvastatin alone could not reduce the collagen expression, as measured by the a‐SMA and COL1A1 protein expression (Supporting information Figure S18). Based on these findings, it seems reasonable that the poor efficacy of atorvastatin alone in preventing PF in mice. Combined treatment, however, suppressed BLM‐induced LDLR deficiency, while atorvastatin alone or alirocumab alone only caused a slight increase. In fact, statins can not only enhance the expression of LDLR, but also that of PCSK9, which specifically degrades LDLR, suggesting combined treatment can maximize and sustain LDLR levels. Therefore, it seems reasonable to treat PF with a combination of statins and PCSK9 inhibitors. We investigated the dose‐response relationship of atorvastatin alone in a BLM‐induced mouse model of PF, and found that a higher dose of atorvastatin (10 mg/kg/d) exhibits no benefits (Supporting information Figure S19). In our animal experiments, we used 5 mg/kg of atorvastatin combined with anti‐PCSK9 to increase LDLR levels. A normal atorvastatin dosage in humans is 10–80 mg per day, which is approximately equivalent to 2.1–16.4 mg/kg in mice. We think that the dosage used in our study is appropriate. An increasing body of evidence has indicated that statins may have a beneficial effect on clinical outcomes in PF and even in COVID‐19.^23^, ^36^, ^37^, ^38^ By searching all the literature investigating the association of statins with PF, including 7 clinical studies^23^, ^24^, ^39^, ^40^, ^41^, ^42^, ^43^ and 18 experimental studies,^24^, ^36^, ^44^, ^45^, ^46^, ^47^, ^48^, ^49^, ^50^, ^51^, ^52^, ^53^, ^54^, ^55^, ^56^, ^57^, ^58^, ^59^ we found that almost all studies trends to indicate that the statins are protective for PF (Supporting information Table S3). It is believed that lipophilic statins are taken up faster by PF lung tissue through passive diffusion than through active uptake.^60^ Moreover, it is hardly for hydrophilic statins to enter other organs.^61^ The human protein atlas shows PCSK9 is enriched in hepatocytes and lung alveolar cells (https://www.proteinatlas.org/ENSG00000169174‐PCSK9). Moreover, Suh et al. report that PCSK9 is highly expressed in mouse lungs.^62^ Xu et al. report human lung A549 cells express PCSK9 as well.^63^ Furthermore, we found that atorvastatin combined with alirocumab administration effectively restored lung LDLR levels. Based on these results, we concluded that the in vivo effects of treatment depend on the lung mechanisms to a great extent. Further, as the liver is also a source of PCSK9, we will evaluate the contributions of hepatic PCSK9 to PF. As statin is a lipid mediator by controlling the LDL, LDLR, PCSK9 levels. However, none of these studies focus on the regulation and the roles of LDL–LDLR‐PCSK9 pathways in PF. It is the first study to demonstrate that lung fibrosis can be alleviated with a pharmacological intervention that targets LDL–LDLR metabolism by atorvastatin combined alirocumab treatment.

Several mechanisms may explain why reduced LDLR results in greater susceptibility to PF. We think cholesterol‐dependent and independent pathways are two underlying mechanisms. A number of studies have reported that cholesterol could induce cell apoptosis, inflammation, EMT and fibrosis.^64^, ^65^, ^66^, ^67^ Besides, LDLR may directly regulate the fibrotic pathways in a cholesterol‐independent manner. Studies have shown that LDLR can bind to DAB2, which acts as a mediator of TGF‐β signal by directly binding to TGFBR1, TGFBR2, SMAD2 and SMAD3.^68^, ^69^ There are some limitations to our study. First, follow‐up studies are required to clarify whether *Ldlr−*/− mice will spontaneously develop fibrosis during aging. Second, the potential contributions of immune cells are also worthy of further study. Finally, combined treatment with statins and anti‐PCSK9 restores LDLR expression and blocks PF progression in mice, but we did not investigate whether combined treatment can cure established PF, and we did not investigate the clinical value of combined treatment for patients.

In conclusion, this study revealed that abnormal LDL–LDLR metabolism stimulates apoptosis, increases fibroblast‐like endothelial and ATII cells and activates fibroblasts, eventually leading to PF. Additionally, we showed that atorvastatin and alirocumab restore LDLR expression and LDL levels, providing proof‐of‐concept that restoration of LDL–LDLR metabolism by pharmacological agents that target LDL and/or LDLR is a promising therapeutic strategy for fibrotic disorders.

## 4 MATERIALS AND METHODS

**Reagents**. BLM (S1214, Selleck), LDL (L7914, Sigma, USA), LPDS (LP4, Millipore, Billerica, MA, USA), atorvastatin (H20051408, Sigma), alirocumab (NJ08807, Sanofi), Lipofectamine RNAiMax (13778, Life Technologies, Gaithersburg, MD, USA), FITC (46950, Sigma), Alexa Fluor 594 (SCJ4600028, Sigma), DAPI (D8417, Sigma) and Fixable Viability Dye eFluor 506 (65‐0866‐14, Thermo Fisher Scientific) were obtained from indicated manufacturers.

**Commercial assays**. The Sircol Assay Kit (S5000, Biocolor), Annexin V‐Alexa Fluor 488/PI Apoptosis Detection Kit (6592, CST, Massachusetts, USA), Endothelin 1 ELISA Kit (DET100, R&D), TUNEL Kit (11684795910, Roche, Indianapolis, IN), Mouse PCSK9 SimpleStep ELISA Kit (ab215538, Abcam, Cambridge, UK), Human PCSK9 SimpleStep ELISA Kit (ab209884, Abcam), Mouse TGF beta 1 ELISA Kit (ARG80211, Arigo) and Human TGF beta 1 ELISA Kit (ARG80123, Arigo) were obtained from indicated manufacturers.

**Mice**. *Ldlr−*/− and C57BL/6J mice were obtained from Jackson Lab and Shanghai SLAC Laboratory Animal Center, respectively.

**Human specimens**. Patients diagnosed with SSc‐PF at the Shanghai Traditional Chinese Medicine‐Integrated Hospital in 2013 were enrolled. The IPF and control lung samples were obtained as a gift from Yuehai Ke, Jingyu Chen and UGMLC Giessen Biobank. Laboratory tests were conducted to confirm the diagnosis for SSc, SSc‐PF and IPF. Age‐ and gender‐matched healthy individuals were enrolled as controls. Patients or controls who were currently on lipid‐lowering medication or had lipid metabolism disorders were excluded from this study. The exclusion criteria also included obesity, diabetes mellitus, hypertension, atherosclerosis and familiar dyslipidaemia. Plasma lipid levels were measured using an automatic biochemical analyser.

### 4.1 Animal protocols, diets and treatment

Six‐week‐old male WT and *Ldlr−*/− mice were intratracheally instilled with 2.0 mg/kg BLM (diluted in sterile saline) or equal volumes of saline as described previously.^67^ Our and other studies showed that the best age of the mouse for PF model is 6–8 weeks.^70^, ^71^ Mice in this period are susceptible to BLM‐induced PF. Additionally, the TGF‐β1‐induced PF model was constructed by an AdTGF‐β1. *Ldlr−*/− and WT mice were intratracheally instilled with single 5 × 10^8^ PFU of AdTGF‐β1 or equal volume of empty adenoviral vector (AdCtr), and euthanized at day 21 for assessment of fibrosis. Mice used for LDL–LDLR metabolism detection shown in Figure 2 were intratracheally instilled with 2.5 mg/kg BLM (diluted in sterile saline) or equal volumes of saline. Mouse SSc‐PF model was induced by subcutaneous injection of BLM (1 mg/mL, 100 μL) or equal volumes of saline every day as described previously.^72^ Mouse lung samples of TopoI‐CFA and GVHD‐induced PF model were gifts from Jörg H.W. Distler. For lipid detection in the plasma, mice were fasted overnight with access to water ad libitum. To evaluate the effects of alirocumab and atorvastatin on PF, mice were treated with alirocumab (subcutaneous injection, 3 mg/kg/week, 7 days before or 7 days after BLM instillation) and atorvastatin (orally, 5 mg/kg/d, 7 days before or 7 days after BLM instillation), alone or both and last until the 21 days after 4 mg/kg BLM instillation. BALF was obtained by washing the alveoli three times using PBS with 1% FBS. Tissue for histological analysis was fixed and imaged using a Nikon Eclipse 80i microscope (Nikon, Badhoevedorp, Netherlands). PF in the lung sections was scored on a scale from 0 to 8 using the Ashcroft score method.

### 4.2 Cell culture

Lung tissue explants of IPF patients and SSc‐PF patients, as well as discarded normal transplant donor lung tissues, were obtained and used to isolate primary endothelial, ATII and fibroblast cells. Primary human lung endothelial, ATII and fibroblast cells were isolated from PF patients. After FAC‐sorting, primary fibroblasts were cultured in Dulbecco's Modified Eagle Medium (DMEM) supplemented with 10% FBS. The primary human lung endothelial and ATII cells were seeded in Fibronectin‐coated plates and cultured in Dulbecco's Modified Eagle Medium (DMEM) supplemented with 10% FBS. All cells were cultured at 37°C in a 5% CO_2_ humidified incubator. For conditioned medium collection, LDL was first incubated with endothelial or ATII cells for 12 h. Then, the conditioned medium was collected after cells were cultured without LDL for another 48 h. For LDL stimulation, all types of cells were cultured in medium with LPDS.

**BALF, plasma, and tissue preparation**. Mice were sacrificed, blood was collected in anti‐coagulant tubes, and plasma was obtained by centrifugation at 3000 × *g* for 10 min and stored at −80°C until ELISA and blood biochemistry assays. Left lungs were ligated and the alveoli were washed three times using PBS with 1% FBS. Tissue for histological analysis was immersed in 4% paraformaldehyde and then embedded in paraffin, and tissue for qRT‐PCR and western blot analysis was frozen and stored at −80°C.

**Histological analysis**. H&E and Masson's trichrome staining were used to evaluate the severity of inflammation and fibrosis. All of the slides were scored according to the Ashcroft score method as in previous studies.^73^, ^74^ The sections were imaged using a Nikon Eclipse 80i microscope (Nikon) at 400× magnification. PF in the lung sections was scored on a scale from 0 to 8 using the Ashcroft scoring method.

**Collagen measurements**. Total soluble collagen in lung samples was quantified using the Sircol collagen assay (Biocolor, Belfast, UK) following the manufacturer's protocol. The amount of collagen in lung samples was normalized to the total amount of protein, as determined using a BCA Protein Assay Kit (Beyotime, Nanjing, China).

**TUNEL staining**. To label nuclei of apoptotic cells, tissue sections were stained with the TUNEL method using the In‐Situ Cell Death Detection Kit with fluorescein (Roche) following the manufacturer's protocol.

**Transient RNA interference**. Two predesigned siRNA sequences which target human and mouse *LDLR* were synthesized by GenePharma (Shanghai, China). Sequences for *LDLR* siRNAs were as follows:

*si‐LDLR*, 5′‐CCAGCGAAGAUGCGAAGAUAUTT‐3′;

*si‐Ldlr*, 5′‐GGCCAUCUAUGAGGACAAATT‐3′.

A scrambled sequence (5′‐UUCUCCGAACGUGUCACGUTT‐3′) was used as nontargeting control siRNA. Cells were transiently transfected with 100 pmol of siRNA using Lipofectamine RNAiMax (Invitrogen). After 72 h, the cells were collected for further analysis.

**Immunofluorescence**. The lung specimens were fixed in 4% paraformaldehyde and stained with anti‐LDLR (1:2000, ab52818, Abcam), anti‐Collagen I (1:200, 72026, CST) and/or anti‐SP‐C (Human: 1:500, ab40879, Mouse: 1:2000, ab211326; Abcam), anti‐CD31 (1:100, Abcam) and anti‐α‐SMA (1:50, Abcam). Then specimens were incubated with FITC‐conjugated anti‐mouse IgG or Alexa Fluor 594‐conjugated anti‐rabbit IgG (Sigma). The nuclei were stained with DAPI. To determine the fluorescent signal in tissue sections, fluorescent cells in five different high‐power fields from each slide were quantified. The Zen software (ZEISS Microscopy) was used to visualize images and quantify stain intensity, including the co‐localization of two different immunofluorescence signals.

**Flow cytometry and FACS**. The fresh human or mouse lungs were diced and washed with basic DMEM. Then the tissues were digested in a mixture containing 2 mg/mL collagenase I, 1 mg/mL DNase I, 0.05 mg/mL dispase and 0.04 mg/mL elastase at 37°C for 45 min in a water bath. Digestion was stopped by adding FBS to a final concentration of 10%. Cell suspension was passed once through a 100 mm cell strainer (Miltenyi Biotec, Germany) to remove undigested tissues. After lysis with red blood cell lysate for 10 min, the cell suspension was continuously passed through the 70 and 40 mm cell strainer to remove multicellular debris. Cells were then centrifuged at 500 × *g* at 4°C for 10 min, washed once in PBS and resuspended in 2% FACS buffer. Primary cells were sorted and verified as in previous studies.^75^, ^76^ For human lung cell sorting, the single‐cell suspension was incubated with Fixable Viability Dye eFluor 506 (65‐0866‐14, Thermo Fisher Scientific), anti‐CD45‐APC‐Cy7 (368516, Biolegend), anti‐CD31‐PerCP/Cyanine5.5 (303132, Biolegend), anti‐CD326‐BV421 (324220, Biolegend), anti‐CD104‐PE (327808, Biolegend) and anti‐PDGFRa‐ PE/Cy7 (323508, Biolegend) antibodies. For mouse lung cell sorting, the single‐cell suspension was incubated with Fixable Viability Dye eFluor 506 (65‐0866‐14, Thermo Fisher Scientific), anti‐CD45‐APC‐Cy7 (557659, Biolegend), anti‐CD31‐PE (102407, Biolegend), anti‐CD326‐BV421 (118225, Biolegend), anti‐CD104‐Percp‐Cy5.5 (123613, Biolegend) and anti‐PDGFRa‐APC (135908, Biolegend) antibodies. FACS sorting was conducted using a BD FACS Aria II (BD Biosciences) for 30 min on ice. After incubation, cells were washed, resuspended in 2% FACS buffer and analyzed by flow cytometry. FAC‐sorted endothelial cells, epithelial cells and fibroblasts were collected and subjected to mRNA extraction and qPCR analysis.

**Gene expression analysis**. Total RNA was isolated from cells and tissues using TRIzol reagent following the manufacturer's protocol. Synthesis of cDNA with reverse transcriptase was performed using an M‐MLV First Strand Kit (Life Technologies,). Real‐time qRT‐PCR was conducted on a Roche LC480 Real‐Time PCR system (Applied Biosystems) using the SYBR Green‐based method. Primer sequences are provided in Supporting information Table S2. Relative expression levels of mRNA were determined following normalization to β‐actin or GAPDH.

**ELISA analysis**. The protein levels of active TGF‐β1, ET‐1 and PCSK9 in human and mouse samples were measured using the ELISA kits (R&D Systems, Minneapolis, MN) following the manufacturer's instructions.

**Western blot analysis**. Proteins from cell lysates were separated by 10% SDS‐PAGE and transferred to nitrocellulose membranes (Millipore). Membranes were blocked with BSA in TBST buffer for 1 h and then incubated with primary antibodies overnight at 4°C, followed by incubation with HRP‐conjugated secondary antibodies. Protein bands were detected with an ECL kit (Pierce, Rockford, IL, USA). Each experiment was performed in triplicate. Protein expression was normalized against β‐actin (ab6276, Abcam) or GAPDH (ab8245, Abcam). The following antibodies were used in the analyses: anti‐LDLR (10785‐1‐AP, Proteintech), anti‐SP‐C (ab90716, Abcam), anti‐CD31 (ab28364, Abcam), anti‐E‐cadherin (14472, CST), anti‐VE‐cadherin (ab33168, Abcam), anti‐caspase‐3 (sc‐271759, Santa Cruz, CA, USA), anti‐α‐SMA (ab18147, Abcam), anti‐Collagen I (GB11022, Servicebio), anti‐SMAD2/3 (5678, CST) and anti‐p‐SMAD2/3 (8828, CST).

**Cell apoptosis assay**. Cells were transfected with *si‐LDLR* and control siRNA in six‐well plates, and cells were treated with LPDS and LDL in 12‐well plates. After 72 h, cells were harvested, rinsed with PBS and resuspended in 500 μL 1× binding buffer containing 10 μL propidium iodide and 5 μL Annexin V/FITC (Annexin V/FITC kit; Beyotime). After incubated at room temperature for 10–20 min in the dark, the fluorescence of cells was immediately measured using a flow cytometer.

### 4.3 Quantification analysis

**RNA seq**. Total RNA was extracted from mouse lungs and cDNA libraries were constructed using a KAPA RNA HyperPrep kit (Kapa Biosystems, Wilmington, MA, USA) following the manufacturer's protocol. The cDNA libraries were sequenced using an Illumina HiSeq X Ten system (Illumina, USA). The transcriptome data were analyzed by kallisto and Deseq2.

### 4.4 Statistical analysis

Normality was assessed both visually and with the Shapiro–Wilk test. For normally distributed parameters, the independent sample *t*‐test was used if the variance is homogenous, otherwise, a non‐parametric Mann‐Whitney test was used. For the parameters that were not normally distributed, a non‐parametric Mann‐Whitney test was used. To compare the effect of *Ldlr* knockout and evaluate the therapeutic effect of statin atorvastatin and alirocumab on PF, a two‐way Analysis of Variance, along with multiple comparison test was used to evaluate significant differences between groups. A *p*‐value less than.05 was considered significant.

## CONFLICT OF INTEREST

The authors declare no conflict of interest.

## Supporting information

Figures S1–S19

Tables S1–S3
