# Supplementary material for: LDLR dysfunction induces LDL accumulation and promotes pulmonary fibrosis
Source: Clin Transl Med. 2022 Jan 26;12(1):e711. doi: 10.1002/ctm2.711 (PMC8792399; doi:10.1002/ctm2.711)
Supplement: Supplementary file 1 — Figures S1–S19 [file CTM2-12-e711-s002.docx]

**ONLINE DATA SUPPLEMENT FIGURES**

**LDLR dysfunction induces LDL accumulation and promotes pulmonary fibrosis**


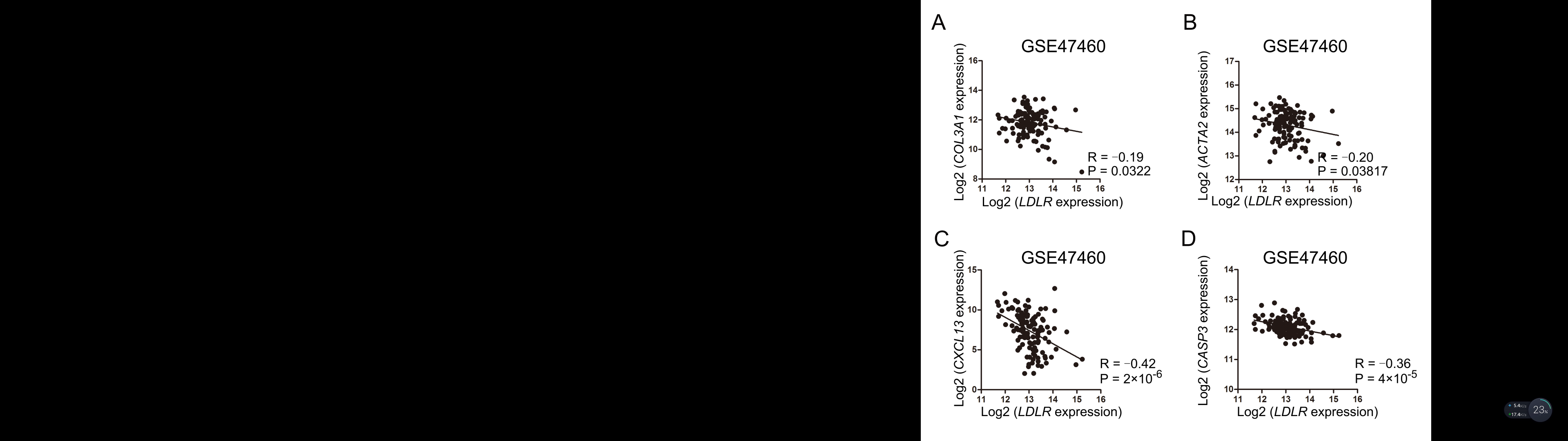


**Supplementary Figure 1 Correlations between LDLR and fibrosis-associated genes. (A–B)** The correlations between LDLR and fibrogenesis-related genes (COL3A1, ACTA2). **(C)** The correlation between LDLR and inflammatory-related genes (CXCL13). **(D)** The correlation between LDLR and apoptosis-related genes (CASP3). The correlation graphs shown in Figures 1A-D were generated from 122 IPF patients of the GSE47460 dataset.


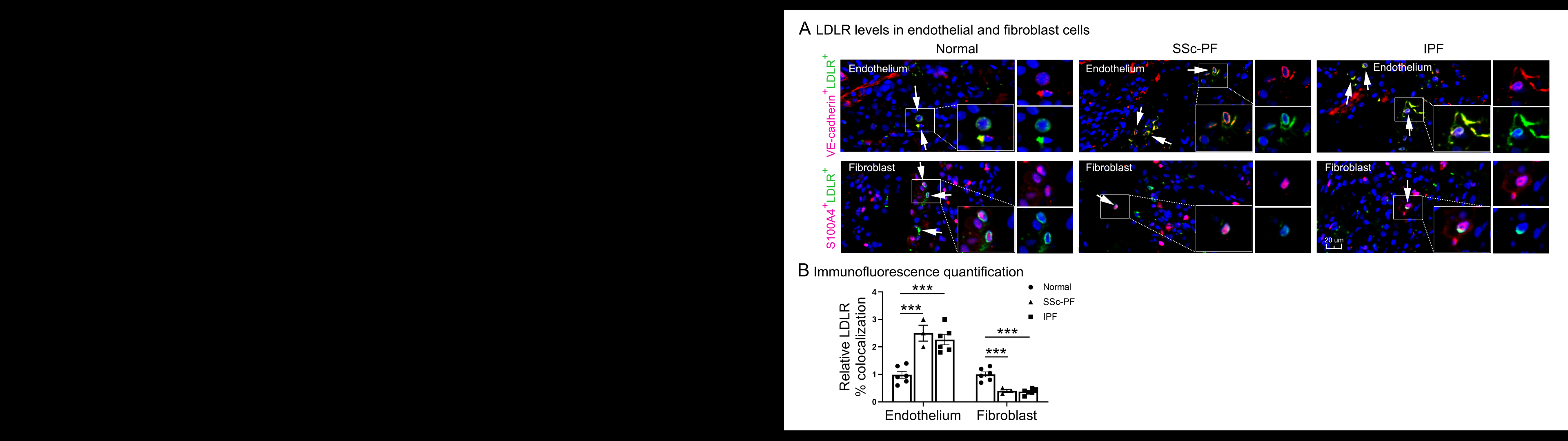


**Supplementary Figure 2 Human lung LDLR levels in endothelial and fibroblast cells by immunofluorescence staining. (A)** Immunofluorescence staining of LDLR and VE-cadherin/S100A4 in lung tissue sections from normal, SSc-PF, and IPF lungs (n = 6, 3, and 6, respectively). The colocalization of LDLR and of each cell marker is expressed as percent double positive area/sum total area of stain for each protein **(B)**. Scale bars: 20 μm. *P < 0.05, **P < 0.01, ***P < 0.001 vs. control.


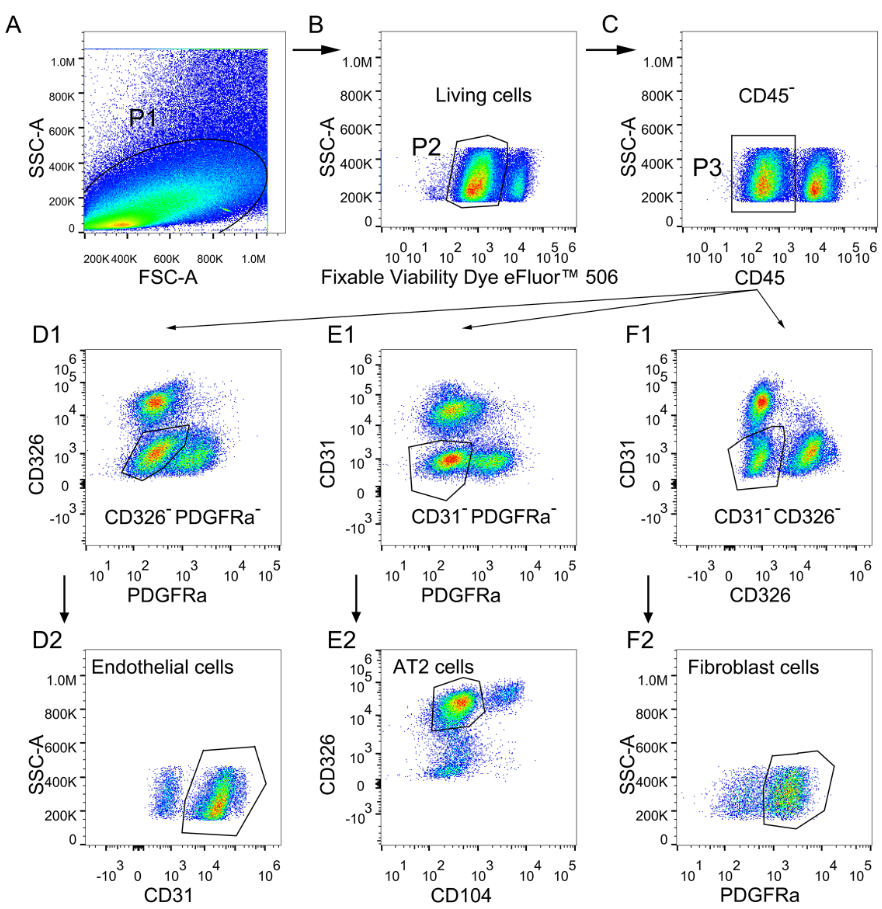


**Supplementary Figure 3** Gating strategy for isolation of endothelial cells, epithelial cells, and fibroblasts from mouse lungs. Total single-cell suspensions were obtained by excluding doublets P1 **(A)**, dead cells P2 **(B)**, and CD45-positive cells P3 **(C)**. Endothelial cells (CD326^-^ PDGFRa^-^ CD31^+^), ATII cells (CD31^-^ CD326^+^ Ter-119^-^ CD104^-^), and fibroblasts (CD31^-^ CD326^-^ PDGFRa^+^) were sorted from the CD45-negative population (**D2, E2**, and **F2**, respectively).


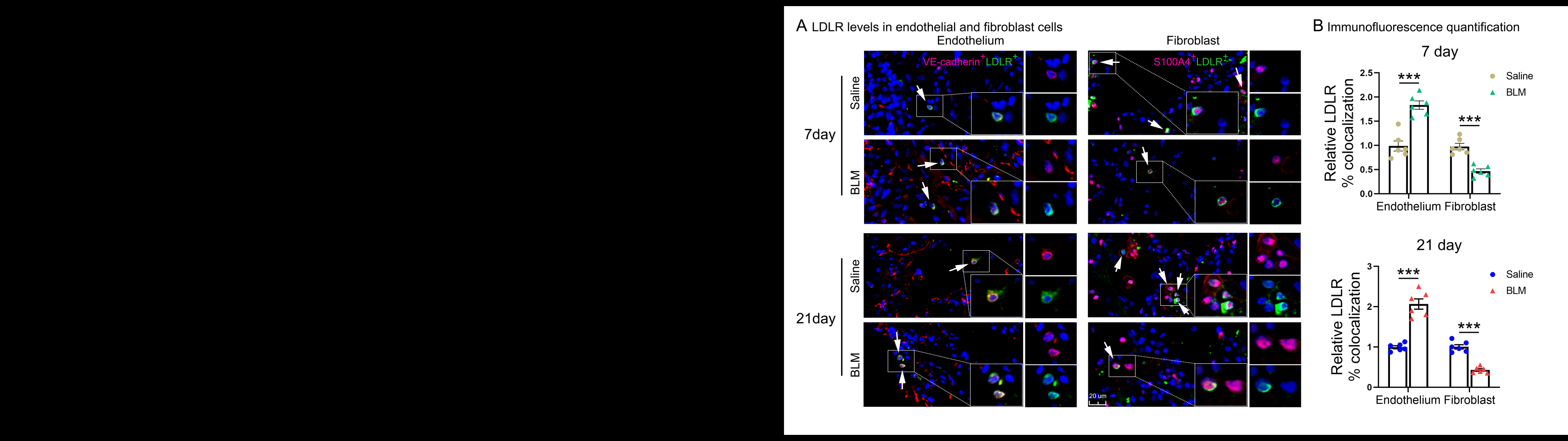


**Supplementary Figure 4 LDLR LDL levels in BLM-induced mouse PF.** **(A)** Immunofluorescence analysis of LDLR protein levels in endothelial and fibroblast cells. **(B)**The colocalization of LDLR and of each cell marker is expressed as percent double positive area/sum total area of stain for each protein. Scale bars: 20 μm. N≥ 6 per group. *P < 0.05, **P < 0.01, ***P < 0.001 vs. control.


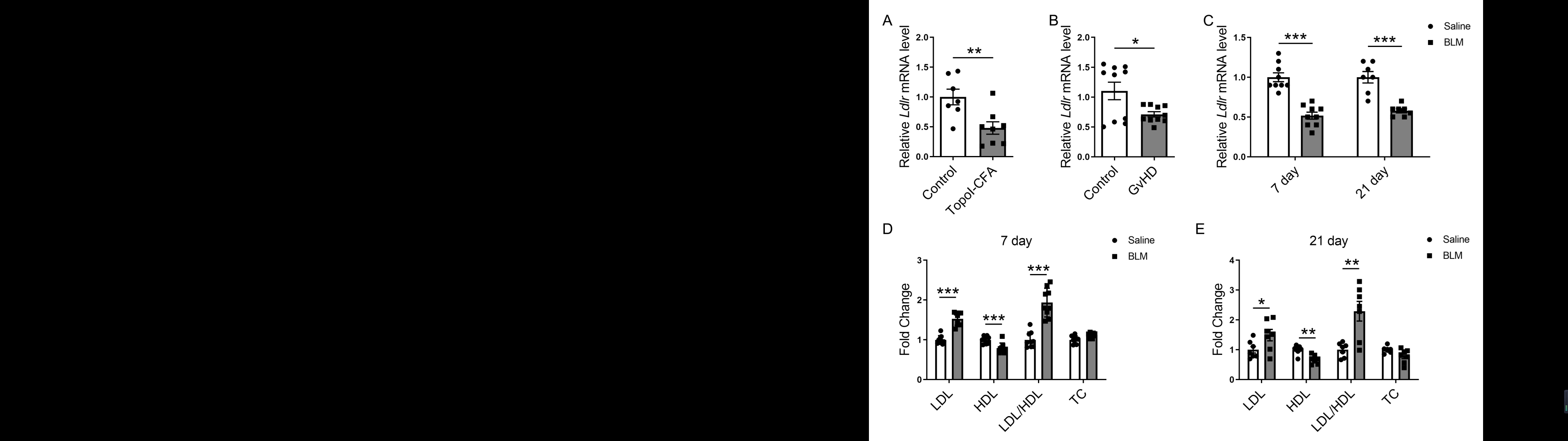


**Supplementary Figure 5 Abnormal LDL–LDLR metabolism in PF mice. (A–B)** *LDLR* mRNA levels in TopoI-CFA- and GVHD-induced PF lungs. **(C–E)** *LDLR* mRNA and lipid levels in BLM-induced SSc-PF mice at 7 and 21 days. *N* ≥7 per group. **P* < 0.05, ***P* < 0.01, ****P* < 0.001 *vs.* control. Data are presented as the mean ± SEM.


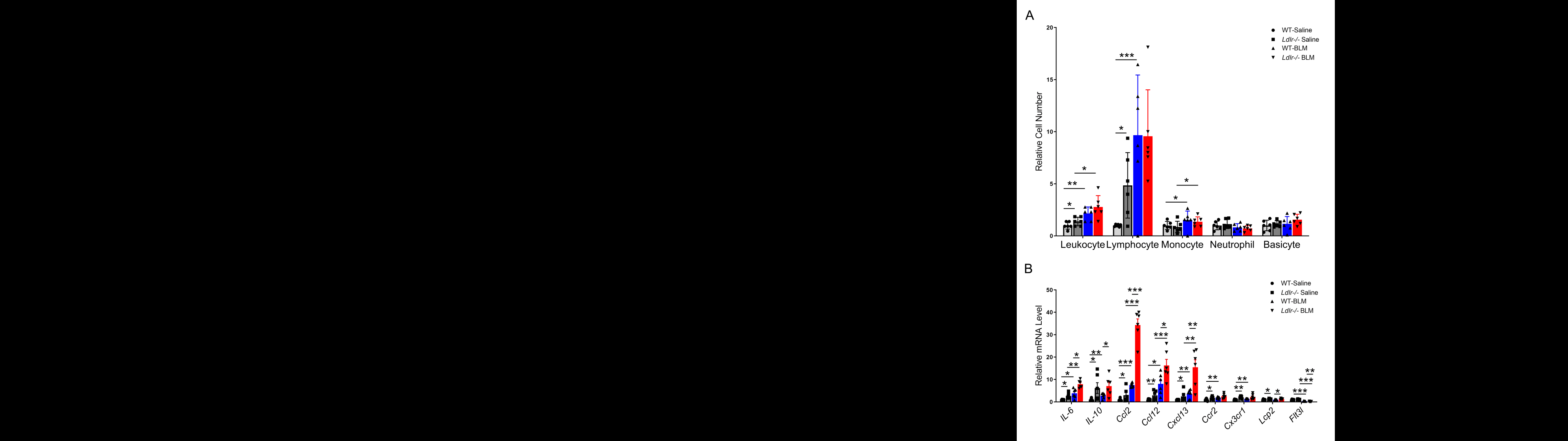


**Supplementary Figure 6 Numbers of inflammatory cells and mRNA levels of inflammation-related genes in *Ldlr* knockout mice. (A)** Cell counts in BALF at baseline and 7 days after BLM treatment. **(B)** *IL-6*, *IL-10*, *Ccl2*, *Ccl12*, *Cxcl13*, *Ccr2*, *Cx3cr1*, *Lcp2*, and *Flt3l* mRNA levels in mouse lungs, as analyzed by qPCR. *N* ≥6 per group. **P* < 0.05, ***P* < 0.01, ****P* < 0.001 *vs.* control. Data are presented as the mean ± SEM.


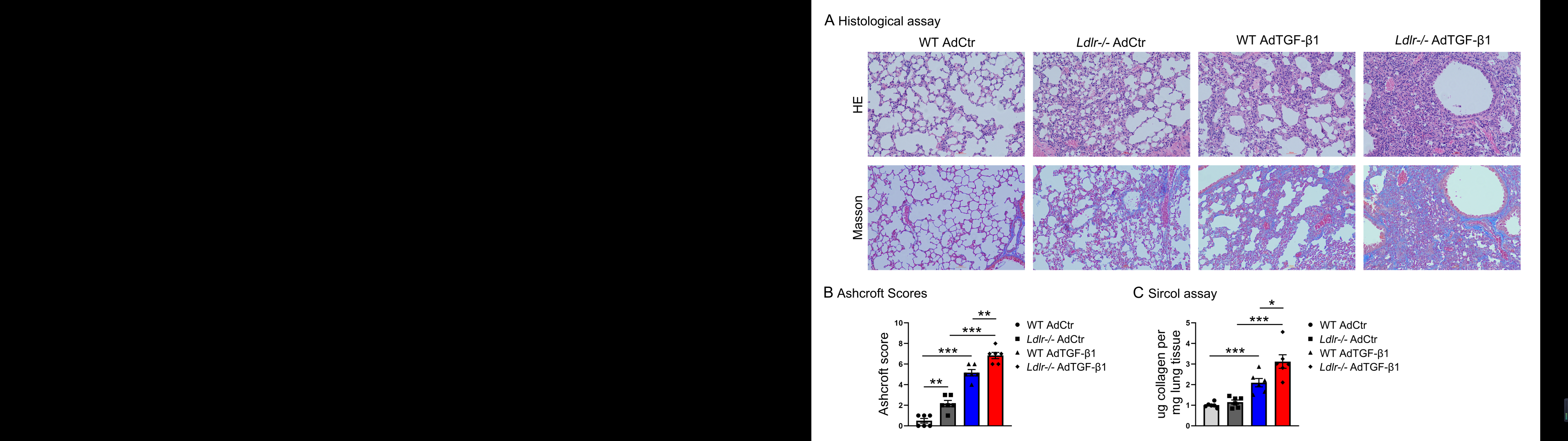


**Supplementary Figure 7 *Ldlr* knockout exacerbated TGF-β1-induced PF. (A–B)** Pulmonary tissue sections were stained with H&E and Masson’s trichrome, and the Ashcroft score was calculated. Scale bar: 50 μm. **(C)** Soluble collagen synthesis in lung homogenate. AdCtr as control. N = 6 per group. *P < 0.05, **P < 0.01, ***P < 0.001 vs. control. Data are presented as the mean ± SEM.


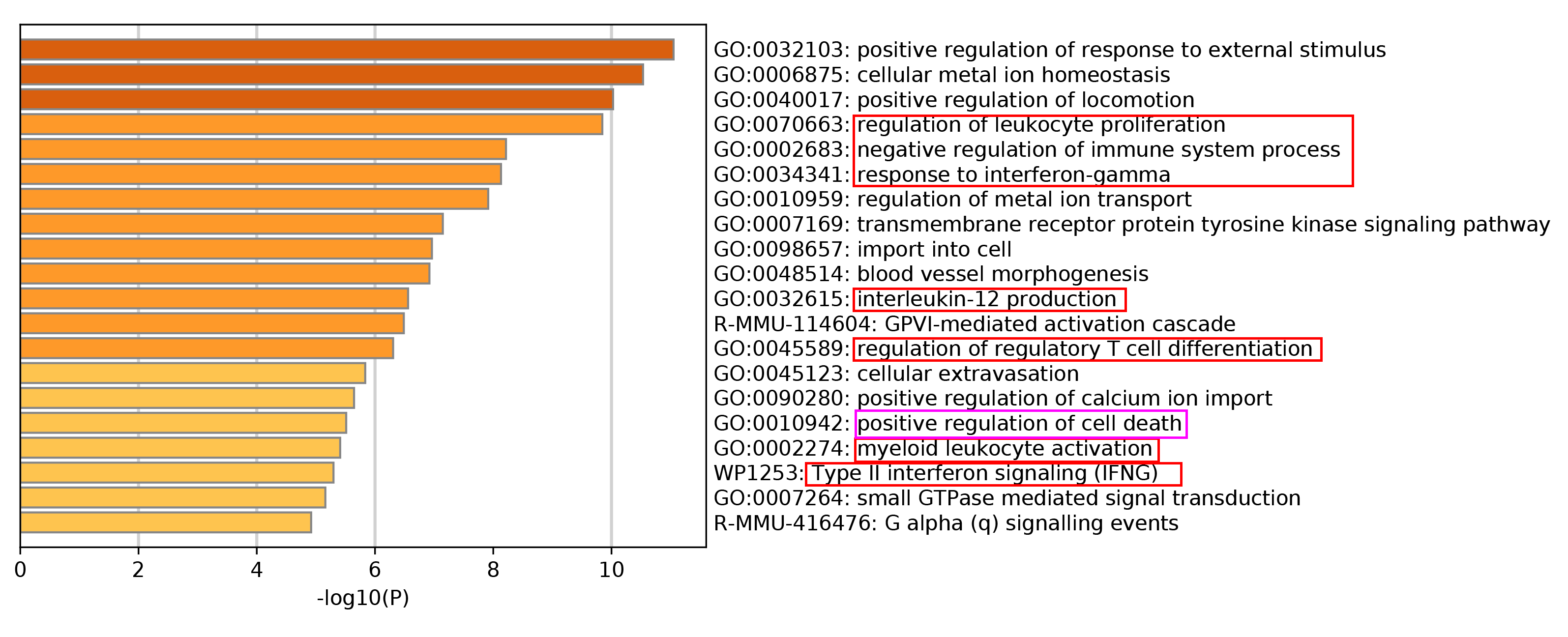


**Supplementary Figure 8** Enrichment analysis of the differentially expressed genes in *Ldlr*-/- mice and WT mice with saline administration. WT-Saline = 6, *Ldlr-/-* Saline = 6.


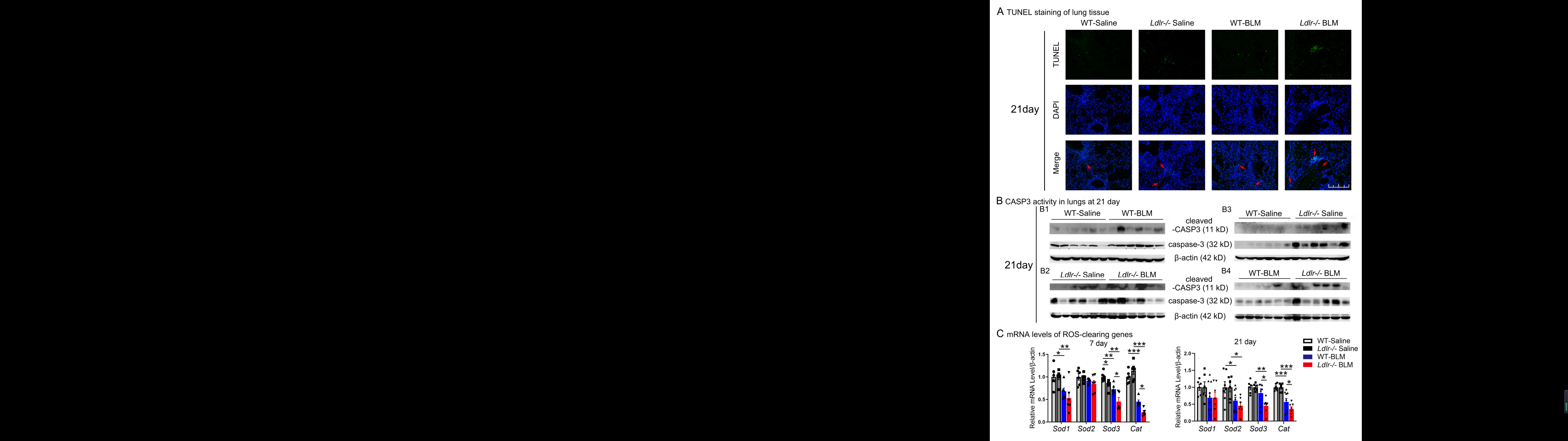


**Supplementary Figure 9 Excessive apoptosis in BLM-treated *Ldlr*-/- mouse lungs at day 21. (A)** TUNEL staining of lung sections of BLM- or saline-treated WT and *Ldlr*-/- mice at 21 days. Scale bar: 250 μm. **(B)** Western blot analysis of cleaved caspase-3 and total caspase-3 levels in the lungs at 21 days after BLM or saline treatment. **B1:** caspase 3 activation in WT mouse lungs; **B2:** caspase 3 activation in *Ldlr-/-* mouse lungs; **B3:** The endogenous levels of cleaved and total caspase 3 in WT and *Ldlr-/-* mice without BLM treatment. **B4:** The levels of cleaved and total caspase 3 in WT and *Ldlr-/-* mice with BLM treatment. **(C–F)** mRNA levels of ROS-related genes in the lungs on days 7 and 21, as analyzed by qPCR. All values are presented as the mean ± SEM of three experiments. β-actin was used as an internal control. mRNA levels were normalized to the saline-treated group. *N* = 6–10 per group). **P* < 0.05, ***P* < 0.01, ****P* < 0.001 *vs.* WT with or without BLM administration.


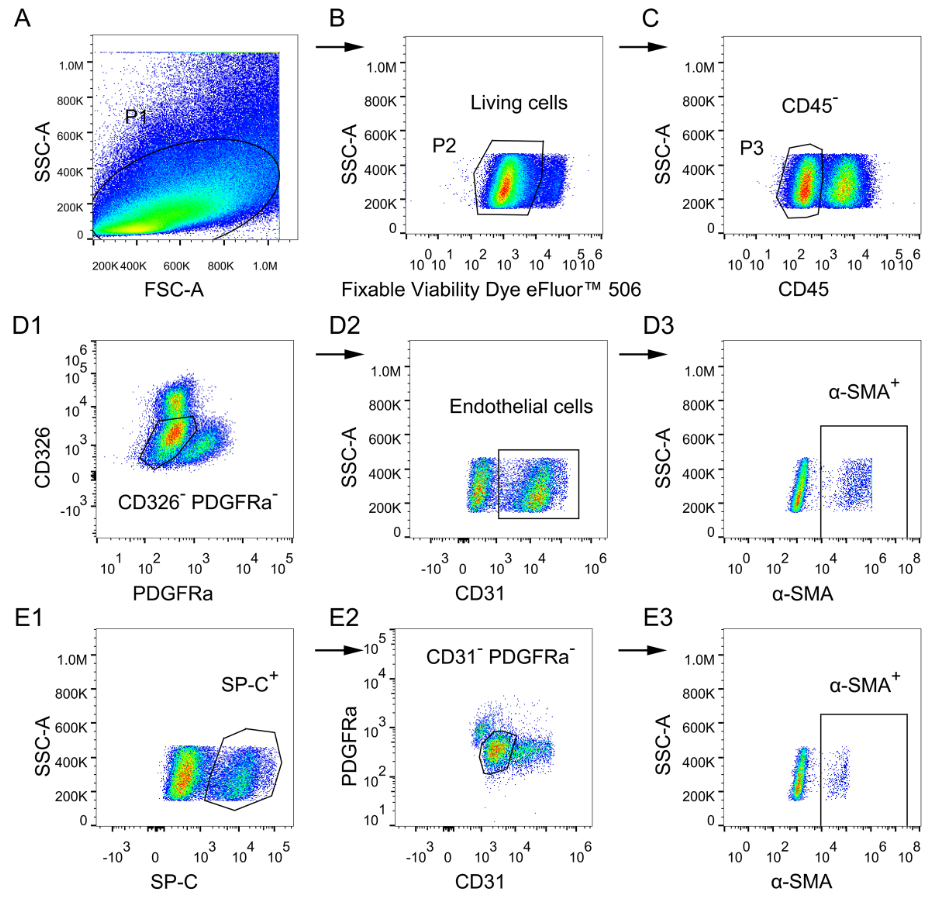


**Supplementary Figure 10 Gating strategy for identification of fibroblast-like endothelial and ATII cells in mouse lungs.** Total single-cell suspensions were obtained by excluding doublets P1 **(A)**, dead cells P2 **(B)**, and CD45-positive cells P3 **(C).** α-SMA^+^ CD31^+^ double positive endothelial cells (CD326^-^ PDGFRa^-^ CD31^+^ α-SMA^+^) and α-SMA^+^ SP-C^+^ double positive ATII cells (CD31^-^ PDGFRa^-^ SP-C^+^) were gated from the CD45-negative population (**D3** and **E3**, respectively).


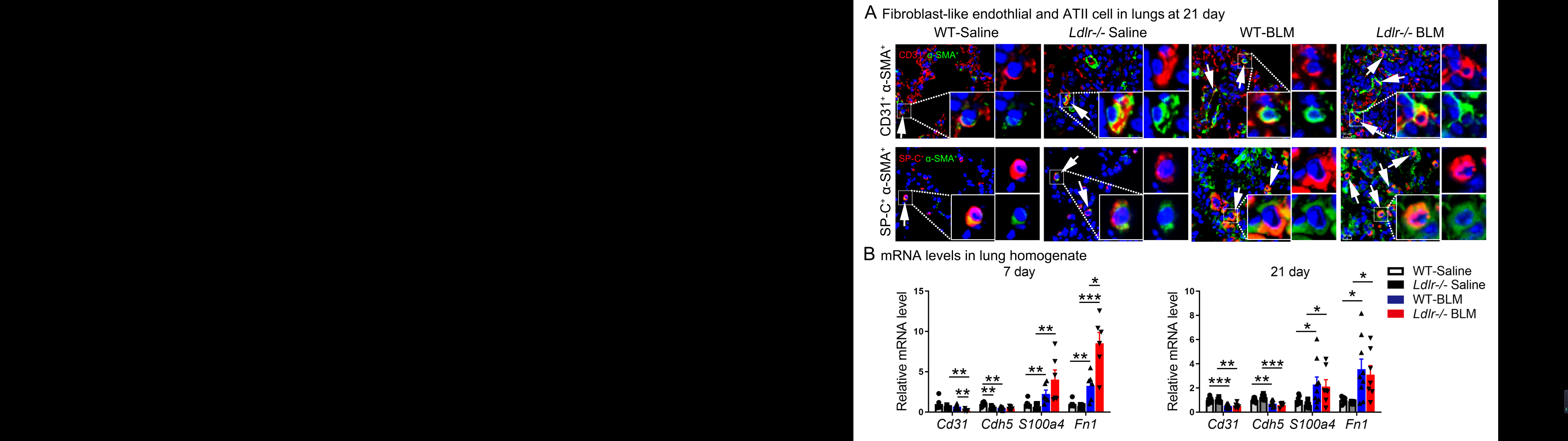


**Supplementary Figure 11 Increased fibroblast-like endothelial and ATII cells in *Ldlr*-/- mouse lungs at 21 day. (A)** Immunofluorescence of α-SMA (green) with the endothelial marker CD31 (red) or ATII cell marker SP-C (red) in mouse lungs at 21 days (*n* ≥ 6 per group). ***P* < 0.01, ****P* < 0.001 *vs.* control, as calculated by the Student’s *t*-test. Data are presented as the mean ± SEM. Scale bar: 50 μm. **(B)** mRNA levels of endothelial, epithelial and mesenchymal cell markers in the lungs, as analyzed by qPCR. mRNA levels were normalized to the saline-treated group. *N* ≥ 6 per group. **P* < 0.05, ***P* < 0.01, ****P* < 0.001 vs. control. Data are presented as the mean ± SEM.


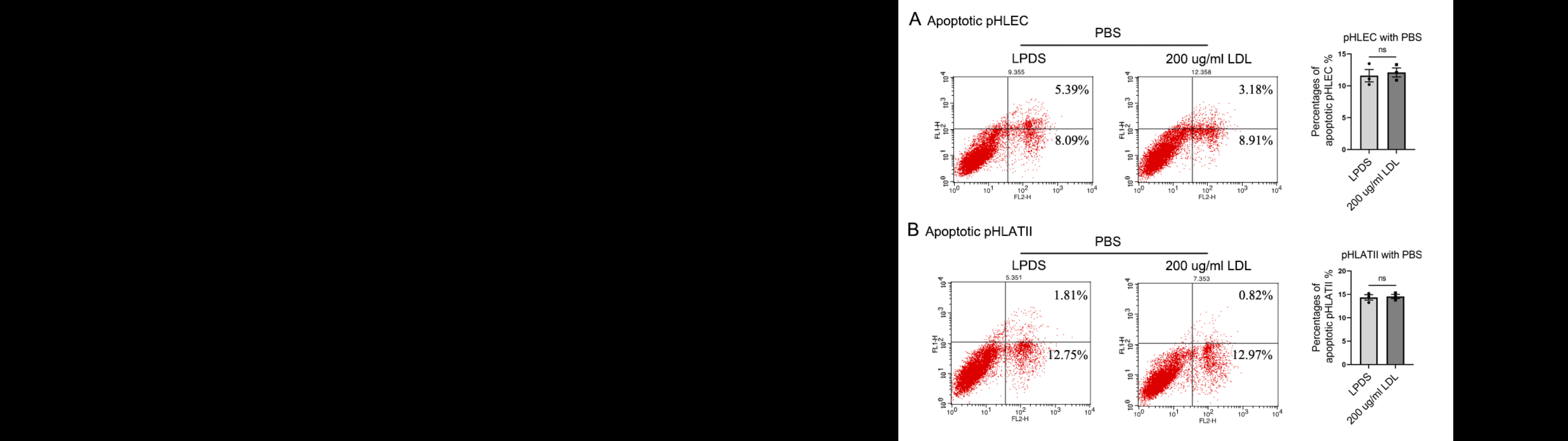


**Supplementary Figure 12 LDL alone had no effect on cell apoptosis without BLM stimulation. (A–B)** Apoptosis in pHLEC and pHLATII cells after LDL stimulation, as analyzed by flow cytometry. **P* < 0.05, ***P* < 0.01, ****P* < 0.001 vs. control. Results are presented as 3 replicates from three independent repeated experiments.


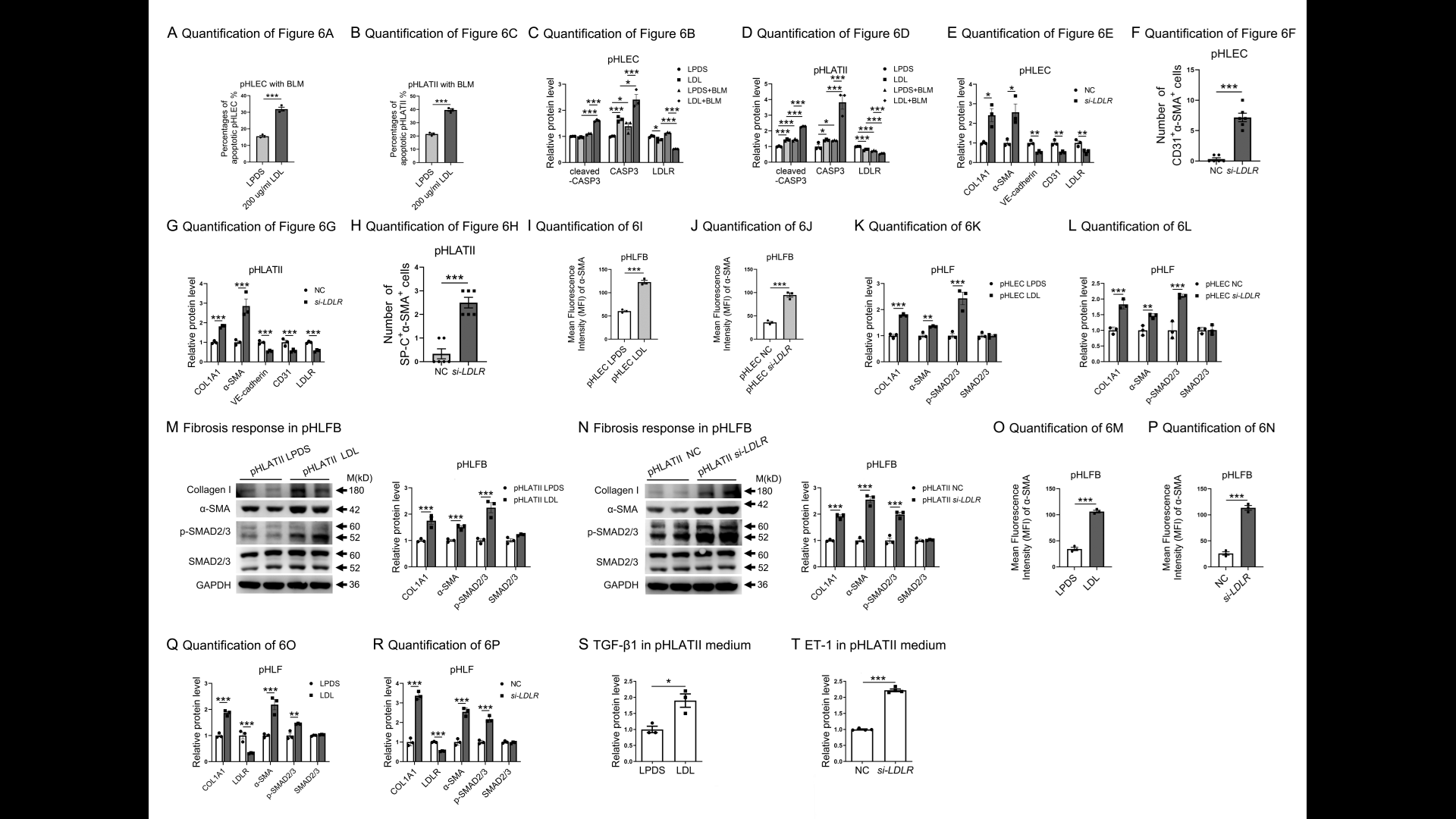


**Supplementary Figure 13 Both high LDL levels and LDLR deficiency led to collagen deposition by activating TGF-β signaling. (A–B)** Apoptotic pHLEC and pHLATII cells after LDL incubation in the presence of BLM. **(C–D)** CASP3 activity in pHLEC and pHLATII cells. **(E–H)** Effects of *si-LDLR* on fibroblast-like changes in pHLEC and pHLATII cells based on western blot and immunofluorescent assay. **(I–J)** Mean Fluorescence Intensity of α-SMA in pHLFB cells. **(K–N)** Quantifications of Collagen, α-SMA, and p-SMAD2/3 levels in pHLFB cells, as analyzed by western blot. **(O–P)** Mean Fluorescence Intensity of α-SMA in pHLFB cells. **(Q–R)** Quantifications of Collagen, LDLR, α-SMA, and p-SMAD2/3 levels in pHLFB cells, as analyzed by western blot. **(S–T)** ELISA analysis of TGF-β1 and ET-1 levels in the culture medium from LDLR-deficient pHLEC and pHLFB cells. LPDS and control siRNA were used as controls. **P* < 0.05, ***P* < 0.01, ****P* < 0.001 vs. control. Data are presented as the mean ± SEM of three independent experiments.


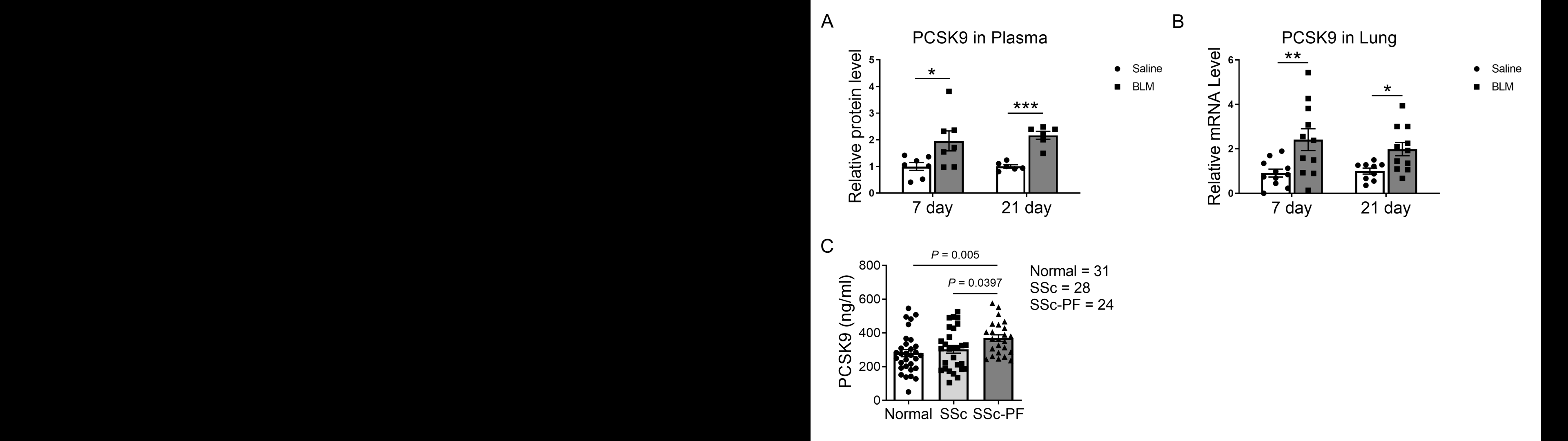


**Supplementary Figure 14 Increased PCSK9 levels in SSc-PF patients and BLM-induced PF mice. (A)** PCSK9 protein levels in BLM-induced PF at 7 and 21 days, as analyzed by ELISA (*n* ≥ 6 per group). **(B)** *Pcsk9* mRNA levels in BLM-induced PF at 7 and 21 days, as analyzed by qPCR (*n* ≥ 6 per group). **(C)** PCSK9 protein levels in SSc-PF patients, as analyzed by ELISA. ***P* < 0.01, ****P* < 0.001 *vs.* control. Data are presented as the mean ± SEM.


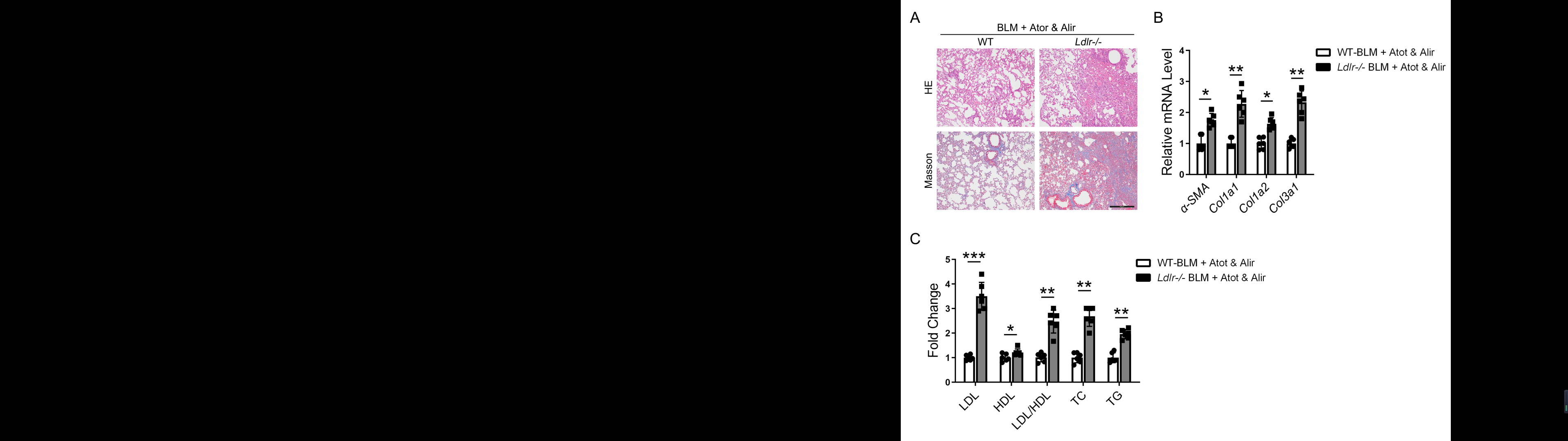


**Supplementary Figure 15 Fibrosis in WT and *Ldlr-/-* mouse lungs upon combined treatment. (A)** Pulmonary tissue sections were stained with H&E and Masson’s trichrome. Scale bar: 500 μm. **(B)** qRT-PCR analysis of fibrosis-related genes in mouse lungs. **(C)** Plasma lipid levels. *N* = 4–6 per group. Data are presented as the mean ± SEM. **P* < 0.05, ***P* < 0.01, ****P* < 0.001 *vs.* WT mouse control.


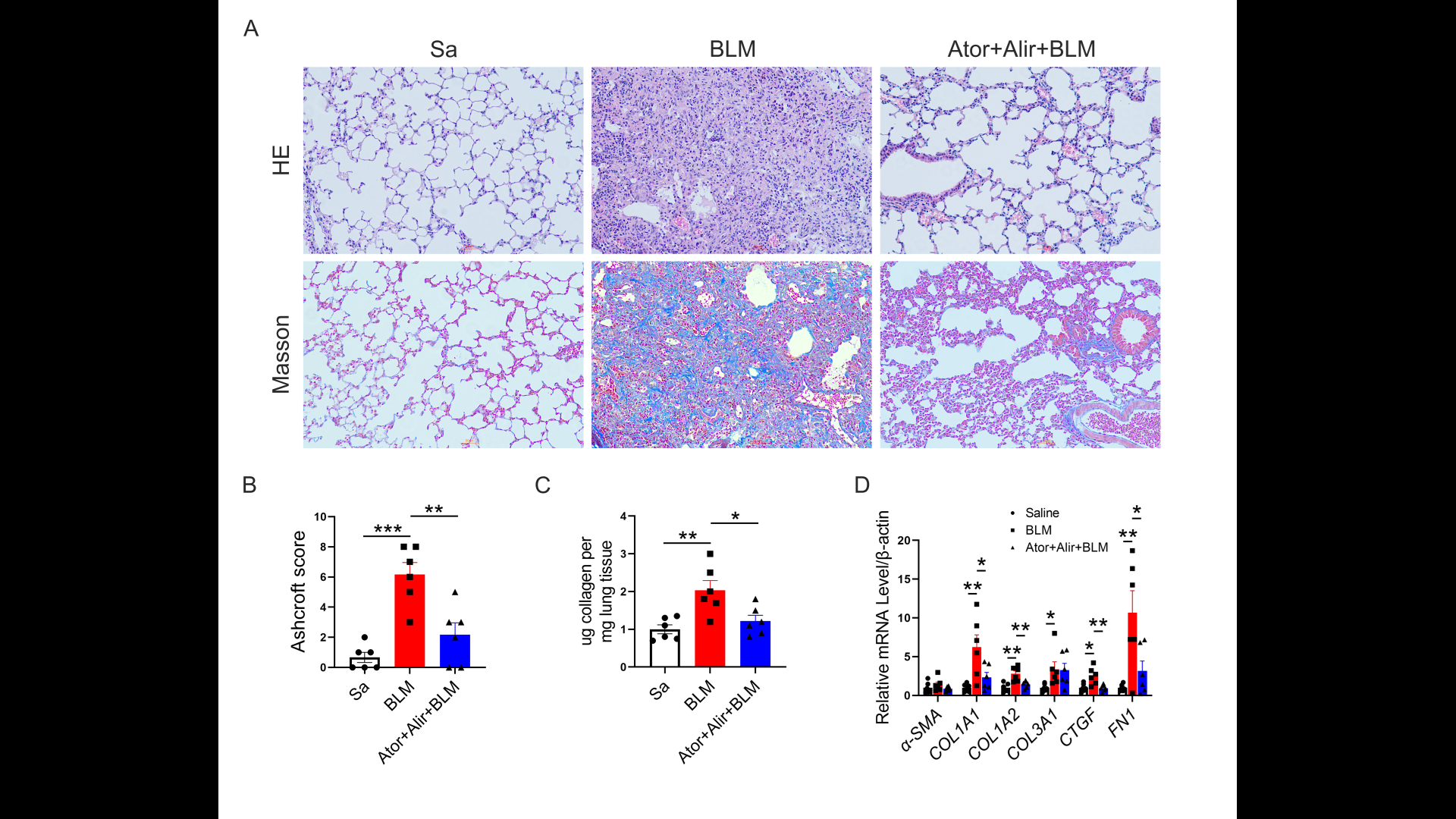


**Supplementary Figure 16 Anti-fibrotic effect of combinational treatment on BLM-induced PF in vivo. (A–B)** Lung tissue sections were stained with H&E and Masson’s trichrome and the Ashcroft score was calculated. Scale bar: 50 μm. **(C–D)** Measurements of ECM content and the expression of collagen genes in lungs of mice receiving different treatments by Sircol assay and qRT-PCR analysis, respectively.
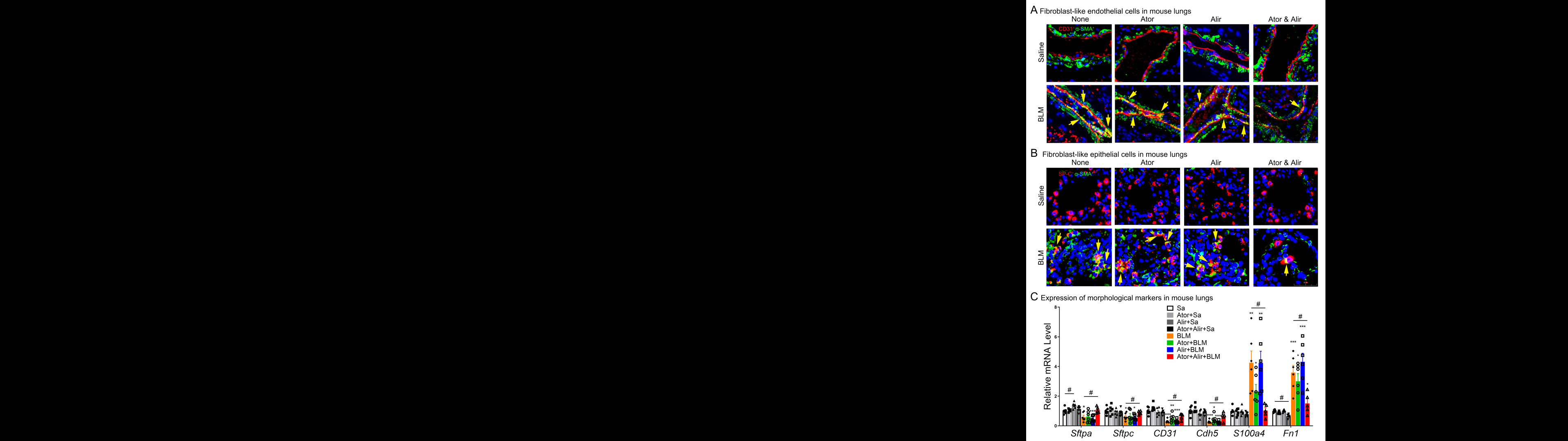


**Supplementary Figure 17 Numbers of Fibroblast-like endothelial and ATII cells in mouse lungs upon combined treatment. (A)** Fibroblast-like endothelial cells were quantified by counting the α-SMA (green) and CD31 (red) double positive cells in mouse lungs. **(B)** Fibroblast-like ATII cells were quantified by counting the α-SMA (green) and SP-C (red) double positive cells in mouse lungs. **(C)** mRNA levels of endothelial, ATII and mesenchymal cell markers in the lungs were quantified by qPCR. *N* ≥ 6 per group. ***P* < 0.01, ****P* < 0.001 *vs.* control. Data are presented as the mean ± SEM. Scale bar: 10 μm.


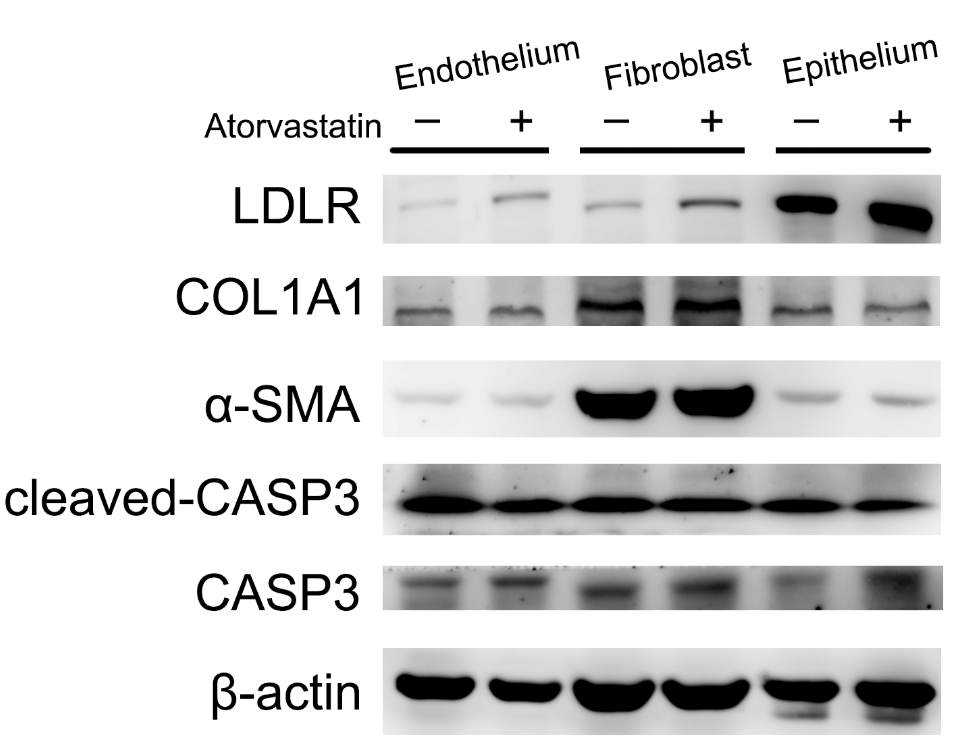


**Supplementary Figure 18 Atorvastatin alone has no effect on cell apoptosis and fibrosis in sorted cells.**


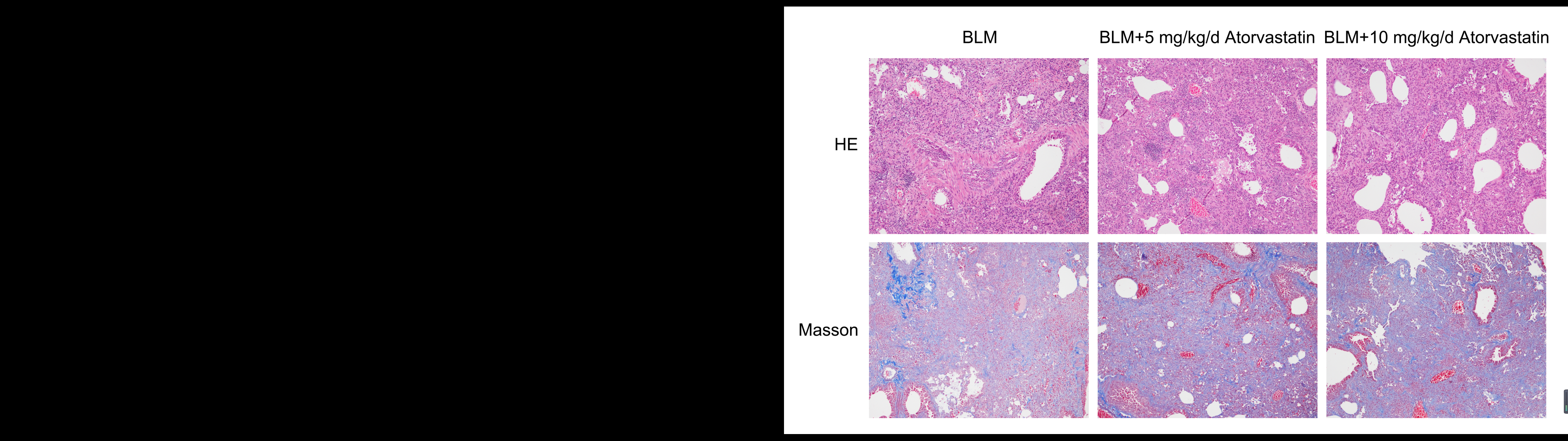
 **Supplementary Figure 19 Atorvastatin alone failed to alleviate PF.** *N* ≥ 6 per group
