# Supplementary material for: LDLR dysfunction induces LDL accumulation and promotes pulmonary fibrosis
Source: Clin Transl Med. 2022 Jan 26;12(1):e711. doi: 10.1002/ctm2.711 (PMC8792399; doi:10.1002/ctm2.711)
Supplement: Supplementary file 2 — Tables S1–S3 [file CTM2-12-e711-s001.docx]

**Supplementary Table 1**

| **Supplemental table 1 Characteristics of SSc, SSc-PF patients and healthy individuals.** | | | | |
| --- | --- | --- | --- | --- |
|  |  |  |  |  |
| **Clinical characteristics** | **SSc** | **SSc-PF** | **Healthy individuals** | **IPF** |
| (mean ± SEM) | (n = 52) | (n = 185) | (n = 1642) | (n = 55) |
| Age (years) | 46.83±11.86 | 50.09±11.99 | 50.07±9.35 | 64.83±16.02 |
| Gender | | | |  |
| Female | 41 | 146 | 1375 | 4 |
| Male | 11 | 39 | 267 | 10 |
| Disease duration (years) | 6.2 ± 1.5 | 6.9 ± 1.1 | / | 1.3 ± 2.2 |
| Classification |  |  |  |  |
| Lc-SSc | 29/52 (55.8%) | 73/185 (39.5%) | / | / |
| Dc-SSc | 23/52 (44.2%) | 112/185 (60.5%) | / | / |
| Autoantibodies |  |  |  |  |
| Anti-topoisomerase antibody (ATA) | 19/41 (46.3%) | 84/130 (64.6%) | / | / |
| Anti-centromere antibody (ACA) | 6/30 (20%) | 10/82 (12.2%) | / | / |
| Anti-nuclear antibody (ANA) | 37/41 (90.2%) | 119/131 (90.8%) | / | / |
| Anti-U1RNP antibody (U1RNP) | 8/30 (26.7%) | 18/101 (17.8%) | / | / |
| Medication history |  |  |  |  |
| Cyclophosphamide | / | 11 (5.9%) | / | 2 (14.3%) |
| Immunosuppressor | 8 (15.4%) | 34 (18.4%) | / | 3 (21.4%) |
| Lipid-lowering drugs, no. (%) | / | / | / | / |
| Number of SSc patients with organ involvement |  |  |  |  |
| Skin involvement | 100% | 100% | / | / |
| Pulmonary fibrosis | / | 100% | / | 100% |
| Diabetes mellitus | / | / | / | / |
| Hypertension | / | / | / | / |
| Atherosclerosis | / | / | / | / |
| Familial dyslipidemia | / | / | / | / |

**Supplementary Table 2**

| **Supplemental table 3 Primer Sequence** | | |
| --- | --- | --- |
| **Primer Name** | **Forward (5' to 3')** | **Reverse (5' to 3')** |
| Mouse *Ldlr* | TCAGACGAACAAGGCTGTCC | CCATCTAGGCAATCTCGGTCTC |
| Mouse *α-Sma* | GTCCCAGACATCAGGGAGTAA | TCGGATACTTCAGCGTCAGGA |
| Mouse *Col1a1* | GGTCCACAAGGTTTCCAAGG | GCTGTTCCAGGCAATCCAC |
| Mouse *Col1a2* | GGACCCGTTGGCAAAGATG | CACCAGGAGGACCAGGAG |
| Mouse *Col3a1* | GAGGAAACAGAGGTGAAAGAGG | CAGCAATGGCAGCAGCAC |
| Mouse *Ctgf* | AAGGACCGCACAGCAGTTGG | AGGCAGTTGGCTCGCATCATAG |
| Mouse *Sftpa1* | GAGGAGCTTCAGACTGCACTC | AGACTTTATCCCCCACTGACAG |
| Mouse *Sftpb* | TTCAAGCCGTGATCCCCAAG | CAGCAGTGCGTCTAGCAGG |
| Mouse *Sftpc* | ATGGACATGAGTAGCAAAGAGGT | CACGATGAGAAGGCGTTTGAG |
| Mouse *Sftpd* | AAGGTCCACGGGGTGAGAA | TTTGCCTTGAGGTCCTATGTTC |
| Mouse *Lpcat1* | GGCTCCTGTTCGCTGCTTT | TTCACAGCTACACGGTGGAAG |
| Mouse *Lpcat2* | CCCTTCGTCCAGCAGACTAC | GCAGCAAAATTATTCCAACCAGT |
| Mouse *Lpcat3* | GACGGGGACATGGGAGAGA | GTAAAACAGAGCCAACGGGTAG |
| Mouse *Lpcat4* | AAACCCGTTCGTGCATGAGTT | CGTTGTGGCATACAGTCTTCCT |
| Mouse *IL-6* | TTGTGCAATGGCAATTCTGA | CTGGCTTTGTCTTTCTTGTTATCT |
| Mouse *IL-10* | GCTGGACAACATACTGCTAACC | ATTTCCGATAAGGCTTGGCAA |
| Mouse *Ccl2* | TTAAAAACCTGGATCGGAACCAA | GCATTAGCTTCAGATTTACGGGT |
| Mouse *Cxcl12* | TGCATCAGTGACGGTAAACCA | TTCTTCAGCCGTGCAACAATC |
| Mouse *Cxcl13* | TTCTCTGTACCATGACACTCTGC | CGTGGAATCTTCCGGCTGTAG |
| Mouse *Ccr2* | ATCCACGGCATACTATCAACATC | CAAGGCTCACCATCATCGTAG |
| Mouse *Cx3cr1* | TACCTTGAGGTTAGTGAACGTCA | CGCTCTCGTTTTCCCCATAATC |
| Mouse *Lcp2* | AGAGGACTTCCTGTCTGTATCAG | TGGACCCTCGATTCTTTCCATC |
| Mouse *Flt3l* | GCCTGGAGCCCAAATTCCTC | GCTGAAGTAACAGTCAGGTGTC |
| Mouse *Pcsk9* | GAGACCCAGAGGCTACAGATT | AATGTACTCCACATGGGGCAA |
| Mouse *β-actin* | GGCTGTATTCCCCTCCATCG | CCAGTTGGTAACAATGCCATGT |
|  |  |  |
| Human *LDLR* | TCTGCAACATGGCTAGAGACT | TCCAAGCATTCGTTGGTCCC |
| Human *β-actin* | CACAGAGCCTCGCCTTTGCC | ACCCATGCCCACCATCACG |

**Supplementary Table 3**

| **1、Effect Of Statins On Pulmonary Fibrosis-Clinical studies** | | | | | | |
| --- | --- | --- | --- | --- | --- | --- |
|  | Type | Number | Results | Odds Ratio (95% Confidence Interval) | P Value | Journal |
| 1 | Clinical Trial | 276 patients received Stains; 348 did not | Statin users: (1) had lower risks of death or 6 MWD decline | HR 0.69; 95% CI 0.48 to 0.99 | p = 0.0465 | Thorax. 2017 Feb;72(2):148-153. |
|  |  |  | (2) lower all-cause hospitalisation | HR 0.58; 95% CI 0.35 to 0.94 | p = 0.0289 |  |
|  |  |  | (3) lower respiratory-related hospitalisation | HR 0.44; 95% CI 0.25 to 0.80 | p = 0.0063 |  |
|  |  |  | (4) lower IPF-related mortality | HR 0.36; 95% CI 0.14 to 0.95 | p = 0.0393 |  |
|  |  |  | (5) No effect on disease progression | HR 0.75; 95% CI 0.52 to 1.07 | p = 0.1135 |  |
|  |  |  | (6) No effect on all-cause mortality | HR 0.54; 95% CI 0.24 to 1.21 | p = 0.1369 |  |
|  |  |  | (7) No effect on death or FVC decline | HR 0.71; 95% CI 0.48 to 1.07 | p = 0.1032 |  |
| 2 | Cohort Study | 6665 ILD patients of a 1.4 million cohort, 26660 controls | No association between current use of statins and risk of ILD | OR 0.99, 95% CI 0.91 to 1.08 | p＞0.05 | Thorax. 2013 Apr;68(4):361-4. |
| 3 | Case-control study | 12 of 50 were taking statins compared with 20 of 100 controls | No association between statins and the diagnosis of ILD | OR 1.13 95% CI 0.30 to 4.24 | p = 0.85 | Respir Med. 2009 Apr;103(4):503-7 |
| 4 | Randomized Controlled Trial | 312 patients received Stains; 749 did not | Annual rate of decline in FVC decreased 50.8 mL/year in statins users at baseline. | 95% CI: –10.9, 112.5 | p = 0.1065 | Respiration. 2018;95(5):317-326. |
| 5 | Stains users in Smoker Cohort | No ILA = 315 (27%) vs. ILA = 66 (38%) | Statin use was positively associated in ILA: (1) All statins | OR 1.62, 95% CI 1.02 to 2.58 | p = 0.04 | Am J Respir Crit Care Med. 2012 Mar 1;185(5):547-56. |
|  |  | No ILA = 280 (24%) vs. ILA = 51 (32%) | (2) Lipophilic statins | OR 1.37 95% CI 0.83 to 2.26 | p = 0.22 |  |
|  |  | No ILA = 33 (4%) vs. ILA =15 (12%) | (3) Hydrophilic statins | OR 4.00 95% CI 1.86 to 8.59 | p < 0.001 |  |
| 6 | Comparative Study | ILD: 1786 statins users vs. 3572 Never users | Statin reduced mortality in ILD patients | OR 0.73 95% CI 0.68 to 0.79 | p = 7×10^-9^ | PLoS One. 2015 Oct 16;10(10):e0140571. |
|  |  | IPF: 261 statins users vs. 522 Never users | Statin reduced mortality in IPF patients | OR 0.76 95% CI 0.62 to 0.93 | P = 0.05 |  |
| 7 | Longitudinal study | 122 oxygen-dependent PF patients | In oxygen-dependent PF, there was no association between statin treatment and survival. | OR 1.13 95% CI 0.81 to 1.57 | p＞0.05 | Respirology. 2016 May; 21(4):705-11 |

| **2、Effect Of Statins On Pulmonary Fibrosis-Experimental studies** | | | | |
| --- | --- | --- | --- | --- |
|  | The Role of statin in Pulmonary Fibrosis | Statin type | Journal | in vitro or in vivo |
| 1 | Protective | Atorvastatin | Life Sci. 2018 Nov 15;213:126-133 | *In vivo* |
| 2 | Protective | Atorvastatin | Biotech Histochem. 2017;92(7):467-480 | *In vivo* |
| 3 | Protective | Simvastatin | Cell Death Dis. 2017 Jun 8;8(6):e2860 | *In vivo* |
| 4 | Protective | Simvastatin | J Cell Mol Med. 2015 Nov;19(11):2647-54. | *In vivo* |
| 5 | Protective | Atorvastatin | Int J Mol Sci. 2013 Dec 16;14(12):24476-91. | *In vivo* |
| 6 | Protective | Pitavastatin | Life Sci. 2013 Dec 18;93(25-26):968-74. | *In vitro* |
| 7 | Protective | Atorvastatin | Eur J Pharmacol. 2013 Nov 15;720(1-3):294-302. | *In vivo* |
| 8 | Protective | Simvastatin | Cell Physiol Biochem. 2013;31(6):863-74. | *In vitro* |
| 9 | Protective | Simvastatin | Acta Physiol (Oxf). 2013 Jun;208(2):191-201. | *In vivo* |
| 10 | Protective | Simvastatin | Pulm Pharmacol Ther. 2014 Feb;27(1):17-28. | *In vivo* |
| 11 | Pathogenic | Pravastatin | Am J Respir Crit Care Med. 2012 Mar 1;185(5):547-56. | *In vivo* |
| 12 | No effect | Simvastatin | Biol Res. 2012;45(4):345-50. | *In vivo* |
| 13 | Protective | Pravastatin | Clin Exp Pharmacol Physiol. 2010 Nov;37(11):1055-63. | *In vivo* |
| 14 | Protective | Atorvastatin | J Card Fail. 2010 Aug;16(8):679-88. | *In vivo* |
| 15 | Protective | Simvastatin | Chin Med J (Engl). 2008 Sep 20;121(18):1821-9. | *In vivo* |
| 16 | Protective | Simvastatin | Am J Respir Cell Mol Biol. 2005 Apr;32(4):290-300. | *In vitro* |
| 17 | Protective | Simvastatin | Am J Physiol Lung Cell Mol Physiol. 2004 Dec;287(6):L1323-32. | *In vitro* |
| 18 | Protective | Lovastatin | Am J Respir Crit Care Med. 1999 Jan;159(1):220-7. | *In vivo* |
